# Evolutionary Transcriptomics of Cancer Development

**DOI:** 10.3390/ijms26115041

**Published:** 2025-05-23

**Authors:** Roman Ivanov, Dmitry Afonnikov, Yury Matushkin, Sergey Lashin

**Affiliations:** 1 Institute of Cytology and Genetics, Siberian Branch of the Russian Academy of Science, Lavrentiev Avenue 10, 630090 Novosibirsk, Russia; ivanovromanart@bionet.nsc.ru (R.I.); ada@bionet.nsc.ru (D.A.); mat@bionet.nsc.ru (Y.M.); 2 Department of Natural Sciences, Novosibirsk State University, 2 Pirogova Str., 630090 Novosibirsk, Russia

**Keywords:** oncogenomics, cancer progression, phylotranscriptomics

## Abstract

Cancer progression is a complex, multi-stage development process characterized by dynamic changes at the molecular level. Understanding these changes may provide new insights into tumorigenesis and potential therapeutic targets. This study focuses on the evolutionary transcriptomics of cancer, specifically analyzing the Transcriptome Age Index (TAI) across different pathological stages. By examining various cancers at four distinct pathological stages, we identify a significant «hourglass» pattern in TAI indices of ductal carcinoma of the breast, bladder carcinoma, and liver carcinoma, suggesting a conserved evolutionary trajectory during tumor development. The results reveal that early and late stages of these cancers exhibit higher TAI values, indicative of more novel gene expression, while intermediate stages show a dip in TAI, reflecting a more ancient evolutionary origin of expressed genes. This «hourglass» pattern underscores the evolutionary constraints and innovations at play during tumor progression. Our findings contribute to the growing body of evidence that evolutionary principles are deeply embedded in cancer biology, offering new perspectives on the dynamics of gene expression in tumors.

## 1. Introduction

The mechanisms underlying the development of cancer have long been studied from a molecular and clinical perspective. Recently, however, there has been a surge of research demonstrating that cancerous tumors can also be analyzed through the lens of gene evolution, focusing on genes that control their progression [1,2,3,4,5,6,7]. By analyzing the origins of genes and pathways associated with malignant tumors, we can infer why certain genes acquire oncogenic properties and how tumor cells develop their characteristic behavior. As a result, the evolutionary analysis of genes associated with cancer has emerged as an increasingly important field, using molecular phylogenetic and comparative genomic approaches to determine how malignant cells acquire their distinctive properties and adapt to different tumor microenvironments, nutrient scarcity, and immune surveillance [8].

One common study approach to the molecular evolution of genes is phylostratigraphic analysis, which maps each gene to its earliest identifiable ancestor on the taxonomic tree. This assigns genes to specific phylogenetic time intervals (phylostrata) in which they are estimated to have originated [9,10]. This method has revealed that genes involved in fundamental cellular processes often date back to ancient evolutionary epochs, reflecting the conservation of key biological functions.

Building on phylostratigraphic analysis, Domazet-Lošo and colleagues [11], as well as Drost, Grosse, and Quint [12], introduced specialized indices to quantify the contribution of genes of different “evolutionary ages” to the active transcriptome. The first metric, the Transcriptome Age Index (TAI), reflects the average evolutionary age of genes expressed under specific conditions. The second, the Transcriptome Divergence Index (TDI), describes the average degree of transcriptome divergence, indicating whether more conserved or more divergent genes are preferentially activated [12].

An example of a successful application of phylotranscriptomic analysis is the study of the evolutionary features of embryogenesis. This method integrates experimental gene expression data with evolutionary age data to reveal patterns of gene activity across embryonic stages. The results of the analysis show that early and late embryonic stages in *Arabidopsis thaliana*, *Danio rerio*, and *Drosophila melanogaster* are predominantly characterized by the expression of evolutionarily less conserved and older genes, whereas intermediate stages show increased activity of more conserved and younger genes [12,13,14]. This pattern aligns with the previously proposed molecular “hourglass model” of embryogenesis [15].

Later, phylostratigraphic analysis was applied in the study of pathological processes, including oncological diseases [10]. It has been shown that some genes involved in cancer development also have ancient evolutionary origins. Further evidence supporting the evolutionary differences in genes involved in tumor progression has been provided by [16], who used phylostratigraphic analysis of co-expression networks in ovarian cancer. They showed that functional modules associated with tumor progression are not uniformly distributed across gene evolutionary ages. Ancient gene clusters were found to regulate key metabolic and signaling pathways essential for basic cellular functions, while younger genes modulate interactions within the tumor microenvironment and influence metastasis and drug resistance mechanisms.

In the context of cancer research, TAI and TDI metrics allow the identification of stage-specific expression patterns in pathological processes. Specifically, they can demonstrate how the balance between the activity of “ancient” and “young” genes shifts in oncological diseases. Despite high tumor heterogeneity, cancers show convergent evolutionary trends toward a limited set of cellular states. Phylostratigraphic analysis suggests that this shift is driven by the activation of ancient genetic programs and the inhibition of genes that emerged during the evolution of multicellularity [17].

This hypothesis was further supported by the phylotranscriptomic analysis of multiple solid tumors, which showed that the transition from normal to malignant cell states involves a shift in gene expression: ancient genes associated with processes originating in unicellular organisms are activated, while genes associated with multicellular regulatory processes are repressed [18].

Thus, phylotranscriptomics extends traditional phylostratigraphic analysis by providing quantitative metrics to study not only the origins of genes but also their functional activity in evolutionary and pathological contexts.

In this work, we build on these concepts by integrating phylostratigraphic approaches, selective pressure metrics based on *d_N_/d_S_* ratios, and RNA-sequencing data from The Cancer Genome Atlas (TCGA). This combined framework allows us to correlate tumor progression dynamics with changes in the evolutionary age and evolutionary conservation of actively expressed genes.

Applying these methods to nine tumor types—liver carcinoma, lung adenocarcinoma, kidney adenocarcinoma, colorectal adenocarcinoma, breast ductal carcinoma, prostate adenocarcinoma, thyroid carcinoma, bladder urothelial carcinoma, and endometrioid carcinoma of the uterus—as well as to corresponding healthy tissues from the same patients, we aim to identify universal patterns linking gene evolutionary age to malignant progression.

## 2. Results

### 2.1. Analysis of the Evolutionary Characteristics of Transcriptomes

To identify potential evolutionary patterns across pathological tumor stages, we employed the Transcriptome Age Index (TAI) and Transcriptome Divergence Index (TDI). In parallel, we performed differential gene expression analysis to compare tumor samples at various stages with healthy tissues.

TAI values were calculated using the *myTAI* software package (see “Methods” section) [19]. For each cancer type, we evaluated TAI profiles across pathological stages of tumor development. The majority of tumor-derived samples exhibited lower TAI values compared to their corresponding “normal” tissue samples. TAI reflects the average evolutionary age of genes with the highest expression levels in a given biological process. Thus, lower TAI values in tumor samples from various tissue types are associated with elevated expression of ancient genes.

**Figure 1:**
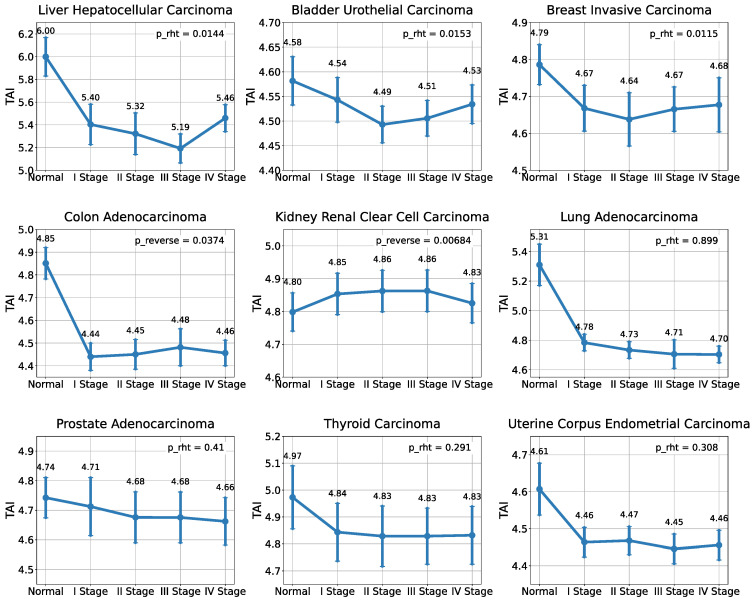
Distribution of TAI values across tumor development stages. Statistically significant hourglass patterns of the Transcriptome Age Index (TAI) are observed in three cancer types: breast ductal carcinoma (BRCA), bladder urothelial carcinoma (BLCA), and hepatocellular carcinoma (LIHC). A statistically significant “reverse hourglass” pattern is observed in colorectal adenocarcinoma (COAD) and clear cell renal carcinoma (KIRC).

When examining TAI value distributions (Table S1) across several carcinoma types—liver carcinoma, bladder urothelial carcinoma, and breast ductal carcinoma—a distinct pattern emerges. Early and late stages of tumor progression (stages I and IV, respectively) show increased activity of evolutionarily recent genes, whereas intermediate stages (stages II and III) show increased activity of genes belonging to more ancient evolutionary groups (Figure 1). These patterns are consistent with the “hourglass” model previously observed in embryonic development, as described by phylotranscriptomics [12,13,14]. The statistical significance of this pattern was confirmed using the ReductiveHourglassTest (*p* = 0.00531 for liver carcinoma, *p* = 0.0337 for bladder carcinoma, and *p* = 0.0107 for breast ductal carcinoma). Notably, TAI distributions in colorectal adenocarcinoma and clear cell renal carcinoma exhibit a significant “reverse hourglass” pattern (*p* = 2.41 × 10^−6^ and *p* = 0.00684, respectively), reflecting an opposite trend. Here, evolutionarily younger genes show higher activity during intermediate stages, followed by the increased expression of ancient genes in late or early stages. In contrast, thyroid carcinoma, lung adenocarcinoma, prostate adenocarcinoma, and uterine corpus carcinoma showed no statistically significant patterns.

**Figure 2:**
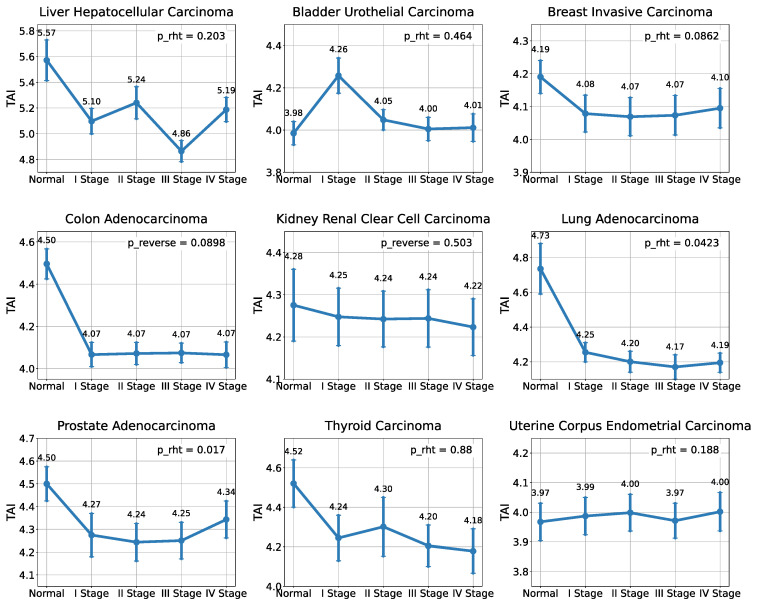
Distribution of TDI values across tumor development stages. Statistically significant hourglass patterns of the Transcriptome Divergence Index (TDI) are observed in two cancer types: lung adenocarcinoma (LUAD) and prostate adenocarcinoma (PRAD).

When examining TDI value distributions (Table S2) in lung adenocarcinoma and prostate adenocarcinoma samples, statistically significant hourglass patterns are also observed (*p* = 0.0423 and *p* = 0.017, respectively) (Figure 2).

### 2.2. **Differential Gene Expression Analysis in Malignant Tumors**

**Figure 3:**
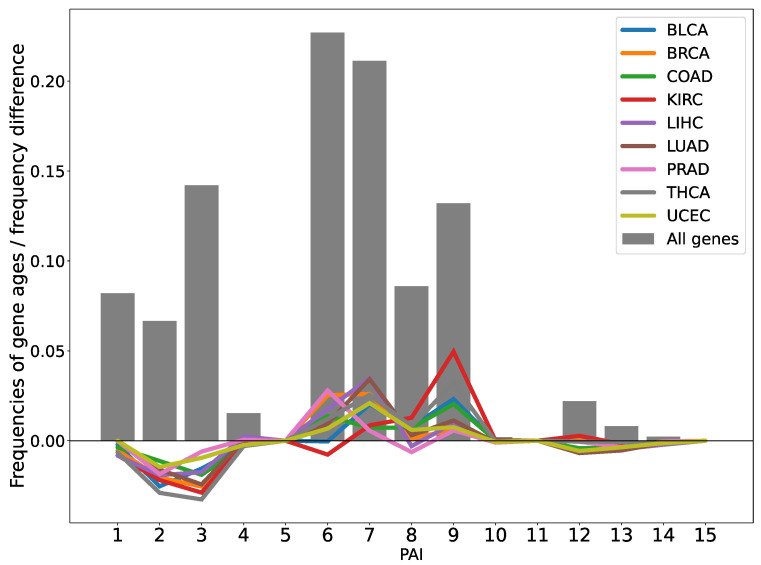
Gray bars represent the distribution of phylostrata among all protein-coding human genes included in the analysis. Colored lines indicate the difference between the frequency of phylostrata in the list of differentially expressed genes (DEGs) for each cancer type and the frequency of the same phylostrata among all human protein-coding genes.

In addition to the TAI/TDI analysis, we performed a differential gene expression analysis of malignant tumors using DESeq2 to identify statistically significant expression changes in samples at each pathological stage compared to corresponding healthy tissue samples (Figure 3). The analysis revealed a significant enrichment of differentially expressed genes belonging to phylostrata PAI (Phylostratigraphic Age Index) = 6, 7, and 9 (Vertebrata, Euteleostomi, and Eutheria, respectively) in human carcinoma tissues compared to the expected distribution of all human genes (*p*-value < 10^−5^) [20]. This result indicates that a greater-than-expected number of novel genes arising in later evolutionary stages undergo significant expression changes during tumorigenesis.

We further analyzed the distribution of log2FC values for DEGs across each phylostratigraphic rank at different pathological stages of tumor progression. The analysis revealed that the median log2FC values for DEGs belonging to the most ancient phylogenetic groups (PAI 1, 2: Cellular Organisms and Eukaryota) trend toward upregulation in most analyzed tumor types and stages (Figures S1–S18). Median log2FC values for genes with PAI = 3–7 are predominantly negative, indicating a tendency toward suppressed expression. In contrast, genes with PAI = 8–14 exhibit the most pronounced expression changes.

**Figure 4:**
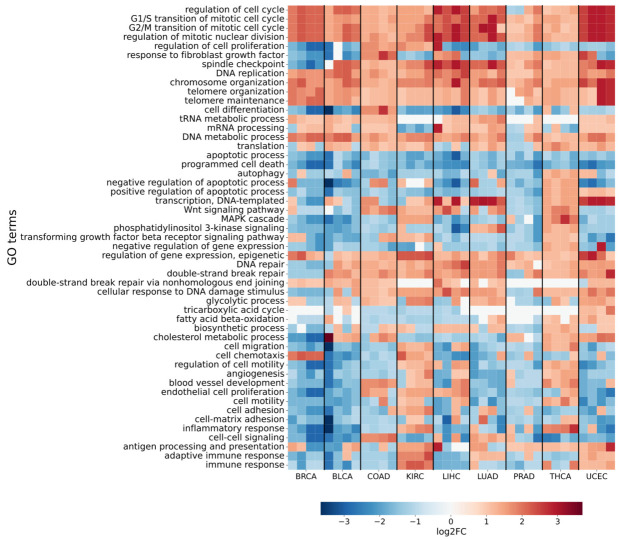
Changes in gene expression levels enriched with GO terms of biological processes associated with cancer hallmarks.

To identify specific functional changes in gene expression during tumor progression, we applied the FoldGO tool [21] (Figure 4). This method detects Gene Ontology (GO) terms enriched in gene sets that show consistent expression shifts (fold change, FC) across tumor developmental stages. Among the results, categories reflecting key aspects of carcinogenesis were of particular interest (Supplementary Table S3).

GO analysis performed with FoldGO revealed that, irrespective of tumor stage or organ, there is a consistent upregulation of genes associated with cell cycle regulation (including G1/S and G2/M transitions and mitosis). This finding aligns with the well-known hallmark of accelerated division in malignant cells [22,23,24,25,26]. In a similar manner, increased expression was observed in genes involved in DNA integrity maintenance, particularly error-prone DNA repair pathways such as nonhomologous end joining (NHEJ), including genes such as *PRKDC* [27,28,29], *MAD2L2* [30,31], and *POLQ* [32,33]. Furthermore, the majority of tumors exhibited elevated expression of genes implicated in translation, transcription, and epigenetic regulation. These changes are likely indicative of the elevated metabolic activity observed in tumors, which necessitates increased protein and RNA synthesis for rapid cell proliferation [23,34,35,36].

At the same time, a decline in the expression levels of genes associated with Gene Ontology (GO) terms related to apoptosis and other forms of programmed cell death is observed in all samples. The suppression of apoptosis is a well-established mechanism through which cancer cells evade programmed death, enabling mutant cells to continue proliferating [22,37,38]. A similar trend is observed for genes associated with intercellular adhesion and cell motility [39,40,41,42].

Notably, during intermediate tumor stages, these broad trends are accompanied by heightened activity of evolutionarily conserved (“ancient”) genes, which drive fundamental cellular processes critical to malignant progression. For instance, genes that demonstrate significant upregulation at intermediate stages (log2FC > 2) include: *AMPK* (PAI 2: Eukaryota), a key kinase in ATP-phosphorylation cascades [43,44,45]; *MSH2* (PAI 2: Eukaryota), responsible for the mismatch repair of nucleotides [46]; *PKM2* (PAI 2: Eukaryota), a pyruvate kinase catalyzing aerobic glycolysis [47,48,49,50]; and *IDH2* (PAI 1: Cellular Organism), an isocitrate dehydrogenase driving the accumulation of the oncometabolite 2-hydroxyglutarate [51,52,53] (PAI 1: Cellular Organism). Simultaneously, genes associated with maintaining cell population balance, such as *SPNS2* (PAI 6: Vertebrata) [54,55] and *STAT5B* (PAI 6: Vertebrata) [56,57], as well as xenobiotic response genes like *CYP3A4 *(PAI 7: Euteleostomi) [58,59] and *GLYAT *(PAI 9: Eutheria) [60,61,62] and ***UGT1*** family glucuronosyltransferases (PAI 7: Euteleostomi) [63,64] show down-regulation in the intermediate tumor stages.

## 3. Discussion

A comprehensive analysis of phylotranscriptomic indices across 5057 carcinoma tissue samples at various pathological stages and 498 corresponding healthy tissue samples from TCGA reveals a correlation between the evolutionary age of genes and their expression changes during tumor development. Our approach leverages the Transcriptome Age Index (TAI), a metric quantifying the weighted evolutionary age of expressed genes across clinical stages. Our observations of consistently lower TAI values in carcinomas compared to healthy tissues align with the atavistic hypothesis of cancer, which posits that malignant transformation involves the reactivation of evolutionarily ancient programs [8,17,65,66,67,68]. This model further suggests that, under conditions of carcinogenic stress or mutation, cells undergo a reversion to “ancient” molecular phenotypes [5,18,69], which prioritize proliferation and survival, similar to unicellular organisms [2,10]. Consequently, tumors display population dynamics broadly similar to that of unicellular life, including high adaptability and rapid clonal expansion [3,5]. This reversion to ancestral programs may enable cancer cells to thrive in the hypoxic, nutrient-poor tumor microenvironment, effectively bypassing the regulatory constraints imposed by multicellularity. Our findings further demonstrate that genes originating at later evolutionary stages (PAI 6: Vertebrata; PAI 7: Euteleostomi; PAI 9: Eutheria) are significantly overrepresented among differentially expressed genes (DEGs) in carcinomas compared to healthy tissues. While the lower TAI suggests a global transcriptional “aging” of tumors toward ancient programs, the enrichment of younger genes in DEGs highlights that novel genetic innovations acquired during vertebrate and mammalian evolution also play outsized roles in cancer progression. The distribution of expression changes across phylostrata further clarifies this tension. Ancient genes (PAI 1: Cellular Organisms, PAI 2: Eukaryota) tend toward upregulation, reactivating deeply conserved pathways linked to proliferation and stress response—processes essential for unicellular survival. Conversely, metazoan genes (PAI 3–7: Multicellular Organisms to Euteleostomi) are broadly suppressed, reflecting their incompatibility with tumor plasticity.

The atavistic hypothesis has previously been discussed in the context of tumor gene expression as a reversion to a “unicellular-like” state [17,18]. However, a thorough examination of the values of the Transcriptome Age Index uncovers a previously unrecognized complexity: tumor progression involves a dynamic interplay between ancient and evolutionarily younger genes, characterized by a distinctive “hourglass” pattern in TAI distributions across specific cancers (e.g., liver adenocarcinoma, bladder carcinoma, breast ductal carcinoma). This parallels the developmental hourglass model, in which mid-stages are dominated by ancient, highly conserved genes, suggesting that tumors transiently reactivate ancestral programs before recruiting newer genes for advanced malignancy. This challenges the paradigm of a unidirectional regression to unicellularity, proposing instead an alternation between ancestral survival programs and newer genes enabling advanced malignancy.

The regression to “ancient” phenotypes in mid-stages during tumor progression (e.g., stage II–III carcinomas) directly corresponds to core hallmarks of cancer, including sustained proliferative signaling, genome instability, and deregulated cellular energetics (Warburg effect) [23]. These ancient pathways prioritize rapid proliferation and stress adaptation, mirroring strategies employed by early eukaryotes under resource-limited conditions. However, this reversion to “ancient” states may carry inherent evolutionary trade-offs: while reactivating ancient genes enhances survival and growth, it also reintroduces vulnerabilities tied to genomic instability (e.g., mismatch repair deficiencies) or metabolic dependencies. For instance, mismatch repair deficiencies—a hallmark of ancient replication systems—create therapeutic opportunities via synthetic lethality (e.g., PARP inhibitors), as observed in BRCA-mutated cancers [70]. Similarly, the activation of aerobic glycolysis, an evolutionarily conserved energy pathway, increases the susceptibility to PKM2 modulators in disrupting tumor metabolism [71,72,73]. The simultaneous downregulation of vertebrate-specific genes involved in cell population balance—such as xenobiotic detoxification (*CYP3A4*, *UGT1*) and intercellular signaling (*SPNS2*, *STAT5B*)—further reflects a regression to autonomous, unicellular-like behavior [54,55,56,57,60,61,62].

In contrast to mid-stage dynamics, advanced tumors exhibited increased TAI values driven by the activation of evolutionarily younger pathways associated with microenvironment remodeling and metastasis. These include angiogenesis, invasion, immune evasion, and extracellular matrix reorganization—processes requiring multicellular coordination [74].

The increase in TAI values at late-stage pathological tumor development can be explained by the reactivation of evolutionarily younger genes that promote aggressive growth, invasion, and metastasis. These genes encompass those implicated in angiogenesis, immune suppression, and metabolic reprogramming. Specifically, genes such as *STC1* (PAI 6: Vertebrata), *VCAN* (PAI 7: Euteleostomi), and *SPP1* (PAI 9: Eutheria)—key regulators of such hallmarks of cancer as angiogenesis and metastasis in tumors [75,76,77,78,79,80,81,82]. These genes, upregulated >3.5-fold in advanced lung, prostate, and breast carcinomas, represent evolutionary innovations critical for metastatic dissemination. These genes represent evolutionary innovations absent in unicellular life, underscoring that metastasis requires multicellularity-derived tools. Their activation suggests that tumors co-opt later-emerged genes to overcome multicellular constraints, such as immune surveillance or stromal barriers. However, this adaptability comes at a cost: the reliance on late-evolving pathways may expose vulnerabilities. For instance, therapies targeting angiogenesis (e.g., bevacizumab) [83] or immune checkpoints (e.g., anti-PD-1) [84] exploit these very mechanisms.

It is important to emphasize that our TAI-based approach captures gene expression dynamics but does not directly assess mutations affecting protein function independently of transcriptional changes (e.g., hyperactivating variants or loss-of-function alleles with unaltered expression) or post-transcriptional regulation by miRNA. Mutation biases, such as hyperactivating KRAS mutations driving proliferation without upregulating mRNA, further complicate interpretation. Tissue-specific effects also emerge; hepatocellular carcinomas showed higher TAI values, likely due to the liver’s inherent metabolic activity. The future integration of somatic mutation data with phylotranscriptomic indices could further disentangle how regulatory and coding genomic alterations collectively shape cancer progression. Also, while our study identifies compelling patterns, the reliance on bulk RNA-seq data may obscure intra-tumor heterogeneity. Single-cell TAI/TDI profiling could resolve whether evolutionary signatures are uniform or confined to subclones.

Thus, our results suggest that tumor progression in at least several cancers does not involve a direct transition to a “unicellular” state but rather reflects a complex dynamic switch between evolutionarily ancient and more recent genes. Our findings suggest that tumors exploit deeply conserved genetic pathways to meet core metabolic and proliferative demands while co-opting younger genes to navigate the complex challenges of tissue invasion, immune evasion, and metastasis.

## 4. Materials and Methods

### 4.1. Expression Data and Tumor Samples

Gene expression data for cancerous tissues were obtained from The Cancer Genome Atlas (TCGA) “https://www.cancer.gov/about-nci/organization/ccg/research/structural-genomics/tcga (accessed on 6 March 2024)”. These data represent transcript counts mapped to each gene, derived from transcriptome sequencing of tumor and matched healthy tissue samples. TCGA and GDC harmonize raw sequencing data through standardized preprocessing pipelines, including batch correction, platform-specific normalization, and transcript quantification using unified workflows (e.g., STAR aligner), ensuring cross-dataset comparability. In this study, we conducted an analysis of gene expression data from the following cancers: hepatocellular adenocarcinoma, clear cell renal adenocarcinoma, colorectal adenocarcinoma, breast carcinoma, prostate adenocarcinoma, thyroid carcinoma, lung adenocarcinoma, bladder urothelial carcinoma, and endometrioid carcinoma of the uterine corpus. The nine cancer types were selected based on their clinical relevance and representation of diverse organ systems. To harmonize pathological stages across datasets, we standardized tumor staging using the American Joint Committee on Cancer (AJCC) TNM classification system [85]. Only samples with definitive staging (Stages I–IV) were included. The analysis was performed on corresponding healthy tissue samples from the same patients (see Table 1 for details). In addition to the gene expression data, clinical observations of patient and tumor characteristics were retrieved from TCGA.

TCGA tissue samples are classified by cancer type and sample type (e.g., primary tumor, normal tissue). Normal adjacent tissue (NAT) samples, defined as histologically normal tissues adjacent to a tumor, are frequently utilized as controls in tumor comparisons. Although, prior studies have demonstrated that gene expression levels in NAT may exhibit slight discrepancies compared to those observed in truly healthy tissues based on the field cancerization and exposure to chronic inflammation [86,87,88,89]. Field cancerization—a phenomenon in which histologically normal tissues surrounding tumors harbor molecular or genetic alterations due to shared exposure to carcinogenic insults—may confound comparisons between tumors and NAT. Similarly, chronic environmental exposures (e.g., chronic inflammation) can induce transcriptomic or epigenetic changes in both tumor and adjacent “normal” tissues, potentially reducing the contrast between disease and control groups. Despite these limitations, we selected NAT samples as controls to mitigate the batch effects and genetic variability that could arise when comparing tumors to healthy tissues collected from distinct protocols or unrelated patients. While this approach prioritizes experimental consistency, we acknowledge that field cancerization and shared environmental exposures may introduce residual biological confounding.

### 4.2. Differential Expression Analysis

Differentially expressed genes (DEGs) were identified using the DESeq2 package [90]. The experimental design aimed to detect genes that exhibited differential expression between tumor samples at specific pathological stages and normal adjacent tissue (NAT) samples from the same patients.

It is important to note that RNA sequencing (RNA-seq) experiments for differential expression analysis frequently involve systematic biases resulting from differences in experimental conditions, sample processing, or sequencing platforms—known as batch effects. The unaccounted batch effects can introduce significant distortions, which complicates the interpretation of results. To mitigate possible technical variability, batch effects were corrected by incorporating the tcga.cgc_case_batch_number variable into the DESeq2 design model.

### 4.3. Phylostratigraphic Analysis

The Phylostratigraphic Age Index (PAI) [91] was developed as a metric for the evaluation of the evolutionary distance of a gene’s origin from the root of the phylogenetic tree. The phylogenetic age of a gene, therefore, is defined as the taxon in which the studied species diverged from its most distant relative where a homolog of the gene was identified. Higher PAI values thus indicate more recent evolutionary origins.

Phylostratigraphic analysis was performed using the Orthoweb software (ver. 1.0.0) “https://orthoweb.sysbio.cytogen.ru/run.html (accessed on 19 March 2024)” [92]. PAI was calculated via the Best Similarity Table KEGG method [93] with a 60% sequence identity threshold for homologs.

In addition to evolutionary age, Orthoweb was used to compute the divergence index (*d_N_/d_S_*) between hominid family members: western gorilla (*Gorilla gorilla gorilla*), Sumatran orangutan (*Pongo abelii*), and common chimpanzee (*Pan troglodytes*).

To analyze the evolutionary age distribution of DEGs and all human protein-coding genes across cancer stages, genes from all DEG lists and the complete human protein-coding gene set were divided into groups by PAI. Contingency tables were constructed to compare the observed proportions of genes in DEG lists against the background distribution derived from the entire protein-coding gene set at distinct cancer stages. Fisher’s exact test was applied to evaluate the statistical significance of differences in the distribution of genes by PAI between DEG lists and the background gene set.

To further assess deviations in PAI distributions within DEG lists from the expected distribution based on the human protein-coding gene repertoire, a chi-square test was performed for each DEG list. Expected frequencies were calculated using the proportions observed in the background gene set. To validate the significance of these deviations, a bootstrap resampling procedure was implemented. Specifically, 100,000 random samples were generated by drawing genes from the background set, with each resampled set matching the size of the corresponding original DEG list. For each resampled set, the chi-square statistic was computed against the expected distribution. The *p*-value for each DEG list was defined as the proportion of bootstrapped samples in which the chi-square statistic exceeded the value obtained from the original DEG list. This approach confirmed that the majority of cancer types exhibited significantly higher chi-square values in their original DEG lists compared to the bootstrapped distributions, reflecting strong statistical evidence for divergent PAI patterns between cancer-associated DEGs and the global protein-coding gene set.

### 4.4. Calculation of Transcriptome Age and Divergence Indices

Phylotranscriptomic indices were calculated using the myTAI package (ver. 0.9.3) [19]. This tool computes the Transcriptome Age Index (TAI) and the Transcriptome Divergence Index (TDI) by integrating external Phylostratigraphic Age Index (PAI) and divergence (DI) data from third-party software. It also assesses the statistical significance of observed patterns. To ensure the proper functionality of the package, d_N_/d_S_ divergence indices were ranked by deciles (10% quantiles). The first decile (Divergence Stratum 1) included the 10% smallest d_N_/d_S_ values, while the tenth decile (Divergence Stratum 10) captured the largest d_N_/d_S_ values (91–100% quantile).

Gene age data, divergence indices (from Orthoweb), and gene expression values for different tumor stages were combined into two datasets for calculating phylotranscriptomic indices. TAI and TDI profiles for each cancer type were visualized and evaluated using the FlatLineTest to assess statistical significance. This method calculates the standard deviation of TAI/TDI profiles and tests their deviation from a flat line (no pattern). When profiles deviated significantly from a flat line, the ReductiveHourglassTest and Reverse Hourglass Test [14] were applied to quantify the statistical significance of visual “hourglass” patterns.

### 4.5. Functional Analysis

FoldGO [21] a web-based tool for functional transcriptome enrichment analysis “http://webfsgor.sysbio.cytogen.ru/ (accessed on 5 February 2024)”, was utilized to identify Gene Ontology (GO) terms that were enriched in gene sets with consistent expression changes across various conditions. FoldGO detects GO terms specifically overrepresented in groups of genes sharing similar fold changes (FC) in expression. The Benjamini–Hochberg correction (FDR < 0.05) was employed to adjust the enrichment significance thresholds for multiple comparisons.

The results obtained from FoldGO were then visualized as a heatmap, where the x-axis represents four tumor stages across nine cancer types and the y-axis lists the enriched GO terms. Each cell displays the median logFC value, reflecting the direction and magnitude of expression shifts within the corresponding functional category at a specific tumor stage. The visualization was implemented using the Python package seaborn (ver. 0.13.2) [94].

## Figures and Tables

**Table 1:** Number of samples with gene expression data retrieved from TCGA.

TCGA_id	Tumor Tissue	I Stage	II Stage	III Stage	IV Stage	NAT
LIHC	Liver adenocarcinoma	255	3	79	3	50
UCEC	Uterine carcinoma	338	53	84	29	35
COAD	Colon adenocarcinoma	168	185	116	22	41
BRCA	Breast carcinoma	215	639	249	13	112
PRAD	Prostate adenocarcinoma	176	174	53	2	52
KIRC	Kidney renal clear cell carcinoma	417	123	124	84	72
LUAD	Lung adenocarcinoma	298	127	84	27	59
BLCA	Bladder urothelial carcinoma	4	130	142	138	18
THCA	Thyroid carcinoma	284	52	112	55	59

## Data Availability

The raw data supporting the conclusions of this article will be made available by the authors upon request.

## Supplementary Materials

The supporting information can be downloaded at: https://www.mdpi.com/article/10.3390/ijms26115041/s1.

## References

[1] Dujon A.M., Aktipis A., Alix-Panabières C., Amend S.R., Boddy A.M., Brown J.S., Capp J., DeGregori J., Ewald P., Gatenby R. (2021). Identifying Key Questions in the Ecology and Evolution of Cancer. Evol. Appl..

[2] Gillies R.J., Verduzco D., Gatenby R.A. (2012). Evolutionary Dynamics of Carcinogenesis and Why Targeted Therapy Does Not Work. Nat. Rev. Cancer.

[3] Greaves M., Maley C.C. (2012). Clonal Evolution in Cancer. Nature.

[4] Maley C.C., Aktipis A., Graham T.A., Sottoriva A., Boddy A.M., Janiszewska M., Silva A.S., Gerlinger M., Yuan Y., Pienta K.J. (2017). Classifying the Evolutionary and Ecological Features of Neoplasms. Nat. Rev. Cancer.

[5] Merlo L.M.F., Pepper J.W., Reid B.J., Maley C.C. (2006). Cancer as an Evolutionary and Ecological Process. Nat. Rev. Cancer.

[6] Mina M., Raynaud F., Tavernari D., Battistello E., Sungalee S., Saghafinia S., Laessle T., Sanchez-Vega F., Schultz N., Oricchio E. (2017). Conditional Selection of Genomic Alterations Dictates Cancer Evolution and Oncogenic Dependencies. Cancer Cell.

[7] Pepper J.W., Scott Findlay C., Kassen R., Spencer S.L., Maley C.C. (2009). SYNTHESIS: Cancer Research Meets Evolutionary Biology. Evol. Appl..

[8] Davies P.C.W., Lineweaver C.H. (2011). Cancer Tumors as Metazoa 1.0: Tapping Genes of Ancient Ancestors. Phys. Biol..

[9] Domazet-Lošo T., Brajković J., Tautz D. (2007). A Phylostratigraphy Approach to Uncover the Genomic History of Major Adaptations in Metazoan Lineages. Trends Genet..

[10] Domazet-Lošo T., Tautz D. (2008). An Ancient Evolutionary Origin of Genes Associated with Human Genetic Diseases. Mol. Biol. Evol..

[11] Domazet-Lošo T., Tautz D. (2010). A Phylogenetically Based Transcriptome Age Index Mirrors Ontogenetic Divergence Patterns. Nature.

[12] Quint M., Drost H.-G., Gabel A., Ullrich K.K., Bönn M., Grosse I. (2012). A Transcriptomic Hourglass in Plant Embryogenesis. Nature.

[13] Cridge A.G., Dearden P.K., Brownfield L.R. (2019). The Developmental Hourglass in the Evolution of Embryogenesis. Evolutionary Developmental Biology.

[14] Drost H.-G., Gabel A., Grosse I., Quint M. (2015). Evidence for Active Maintenance of Phylotranscriptomic Hourglass Patterns in Animal and Plant Embryogenesis. Mol. Biol. Evol..

[15] Irie N., Kuratani S. (2014). The Developmental Hourglass Model: A Predictor of the Basic Body Plan?. Development.

[16] Zhang L., Tan Y., Fan S., Zhang X., Zhang Z. (2019). Phylostratigraphic Analysis of Gene Co-Expression Network Reveals the Evolution of Functional Modules for Ovarian Cancer. Sci. Rep..

[17] Chen H., He X. (2016). The Convergent Cancer Evolution toward a Single Cellular Destination. Mol. Biol. Evol..

[18] Trigos A.S., Pearson R.B., Papenfuss A.T., Goode D.L. (2017). Altered Interactions between Unicellular and Multicellular Genes Drive Hallmarks of Transformation in a Diverse Range of Solid Tumors. Proc. Natl. Acad. Sci. USA.

[19] Drost H.-G., Gabel A., Liu J., Quint M., Grosse I. (2018). myTAI: Evolutionary Transcriptomics with R. Bioinformatics.

[20] Piovesan A., Antonaros F., Vitale L., Strippoli P., Pelleri M.C., Caracausi M. (2019). Human Protein-Coding Genes and Gene Feature Statistics in 2019. BMC Res. Notes.

[21] Wiebe D.S., Omelyanchuk N.A., Mukhin A.M., Grosse I., Lashin S.A., Zemlyanskaya E.V., Mironova V.V. (2020). Fold-Change-Specific Enrichment Analysis (FSEA): Quantification of Transcriptional Response Magnitude for Functional Gene Groups. Genes.

[22] Evan G.I., Vousden K.H. (2001). Proliferation, Cell Cycle and Apoptosis in Cancer. Nature.

[23] Hanahan D., Weinberg R.A. (2011). Hallmarks of Cancer: The Next Generation. Cell.

[24] Otto T., Sicinski P. (2017). Cell Cycle Proteins as Promising Targets in Cancer Therapy. Nat. Rev. Cancer.

[25] Vogelstein B., Kinzler K.W. (2004). Cancer Genes and the Pathways They Control. Nat. Med..

[26] Malumbres M. (2014). Cyclin-Dependent Kinases. Genome Biol..

[27] Chen Y., Li Y., Xiong J., Lan B., Wang X., Liu J., Lin J., Fei Z., Zheng X., Chen C. (2021). Role of PRKDC in Cancer Initiation, Progression, and Treatment. Cancer Cell Int..

[28] Tan K.T., Yeh C.-N., Chang Y.-C., Cheng J.-H., Fang W.-L., Yeh Y.-C., Wang Y.-C., Hsu D.S.-S., Wu C.-E., Lai J.-I. (2020). *PRKDC*: New Biomarker and Drug Target for Checkpoint Blockade Immunotherapy. J. Immunother. Cancer.

[29] Chen Y., Li Y., Guan Y., Huang Y., Lin J., Chen L., Li J., Chen G., Pan L.K., Xia X. (2020). Prevalence of *PRKDC* Mutations and Association with Response to Immune Checkpoint Inhibitors in Solid Tumors. Mol. Oncol..

[30] Li Y., Li L., Chen M., Yu X., Gu Z., Qiu H., Qin G., Long Q., Fu X., Liu T. (2018). MAD2L2 Inhibits Colorectal Cancer Growth by Promoting NCOA3 Ubiquitination and Degradation. Mol. Oncol..

[31] Xu K., Zheng X., Shi H., Ou J., Ding H. (2024). MAD2L2, a Key Regulator in Ovarian Cancer and Promoting Tumor Progression. Sci. Rep..

[32] Wood R.D., Doublié S. (2016). DNA Polymerase θ (POLQ), Double-Strand Break Repair, and Cancer. DNA Repair.

[33] Schrempf A., Slyskova J., Loizou J.I. (2021). Targeting the DNA Repair Enzyme Polymerase θ in Cancer Therapy. Trends Cancer.

[34] Baylin S.B., Jones P.A. (2011). A Decade of Exploring the Cancer Epigenome—Biological and Translational Implications. Nat. Rev. Cancer.

[35] Morgan M.A., Shilatifard A. (2015). Chromatin Signatures of Cancer. Genes Dev..

[36] Truitt M.L., Ruggero D. (2016). New Frontiers in Translational Control of the Cancer Genome. Nat. Rev. Cancer.

[37] Carneiro B.A., El-Deiry W.S. (2020). Targeting Apoptosis in Cancer Therapy. Nat. Rev. Clin. Oncol..

[38] Wong R.S. (2011). Apoptosis in Cancer: From Pathogenesis to Treatment. J. Exp. Clin. Cancer Res..

[39] Janiszewska M., Primi M.C., Izard T. (2020). Cell Adhesion in Cancer: Beyond the Migration of Single Cells. J. Biol. Chem..

[40] Le Bras G.F., Taubenslag K.J., Andl C.D. (2012). The Regulation of Cell-Cell Adhesion during Epithelial-Mesenchymal Transition, Motility and Tumor Progression. Cell Adhes. Migr..

[41] Paul C.D., Mistriotis P., Konstantopoulos K. (2017). Cancer Cell Motility: Lessons from Migration in Confined Spaces. Nat. Rev. Cancer.

[42] Stuelten C.H., Parent C.A., Montell D.J. (2018). Cell Motility in Cancer Invasion and Metastasis: Insights from Simple Model Organisms. Nat. Rev. Cancer.

[43] Herzig S., Shaw R.J. (2018). AMPK: Guardian of Metabolism and Mitochondrial Homeostasis. Nat. Rev. Mol. Cell Biol..

[44] Li W., Saud S.M., Young M.R., Chen G., Hua B. (2015). Targeting AMPK for Cancer Prevention and Treatment. Oncotarget.

[45] Luo Z., Saha A.K., Xiang X., Ruderman N.B. (2005). AMPK, the Metabolic Syndrome and Cancer. Trends Pharmacol. Sci..

[46] Edelbrock M.A., Kaliyaperumal S., Williams K.J. (2013). Structural, Molecular and Cellular Functions of MSH2 and MSH6 during DNA Mismatch Repair, Damage Signaling and Other Noncanonical Activities. Mutat. Res./Fundam. Mol. Mech. Mutagen..

[47] Dong G., Mao Q., Xia W., Xu Y., Wang J., Xu L., Jiang F. (2016). PKM2 and Cancer: The Function of PKM2 beyond Glycolysis. Oncol. Lett..

[48] Wong N., De Melo J., Tang D. (2013). PKM2, a Central Point of Regulation in Cancer Metabolism. Int. J. Cell Biol..

[49] Wu S., Le H. (2013). Dual Roles of PKM2 in Cancer Metabolism. ABBS.

[50] Zhang Z., Deng X., Liu Y., Liu Y., Sun L., Chen F. (2019). PKM2, Function and Expression and Regulation. Cell Biosci..

[51] Ježek P. (2020). 2-Hydroxyglutarate in Cancer Cells. Antioxid. Redox Signal..

[52] Losman J.-A., Kaelin W.G. (2013). What a Difference a Hydroxyl Makes: Mutant IDH, (*R*)-2-Hydroxyglutarate, and Cancer. Genes Dev..

[53] Ward P.S., Patel J., Wise D.R., Abdel-Wahab O., Bennett B.D., Coller H.A., Cross J.R., Fantin V.R., Hedvat C.V., Perl A.E. (2010). The Common Feature of Leukemia-Associated IDH1 and IDH2 Mutations Is a Neomorphic Enzyme Activity Converting α-Ketoglutarate to 2-Hydroxyglutarate. Cancer Cell.

[54] Gu X., Jiang Y., Xue W., Song C., Wang Y., Liu Y., Cui B. (2019). SPNS 2 Promotes the Malignancy of Colorectal Cancer Cells via Regulating Akt and ERK Pathway. Clin. Exp. Pharma. Physio..

[55] Le T.N.U., Nguyen T.Q., Kalailingam P., Nguyen Y.T.K., Sukumar V.K., Tan C.K.H., Tukijan F., Couty L., Hasan Z., Del Gaudio I. (2022). Mfsd2b and Spns2 Are Essential for Maintenance of Blood Vessels during Development and in Anaphylactic Shock. Cell Rep..

[56] Pham H.T.T., Maurer B., Prchal-Murphy M., Grausenburger R., Grundschober E., Javaheri T., Nivarthi H., Boersma A., Kolbe T., Elabd M. (2017). STAT5BN642H Is a Driver Mutation for T Cell Neoplasia. J. Clin. Investig..

[57] Rani A., Murphy J.J. (2016). STAT5 in Cancer and Immunity. J. Interferon Cytokine Res..

[58] Lolodi O., Wang Y.-M., Wright W.C., Chen T. (2018). Differential Regulation of CYP3A4 and CYP3A5 and Its Implication in Drug Discovery. CDM.

[59] Wang F., Zhang X., Wang Y., Chen Y., Lu H., Meng X., Ye X., Chen W. (2023). Activation/Inactivation of Anticancer Drugs by CYP3A4: Influencing Factors for Personalized Cancer Therapy. Drug Metab. Dispos..

[60] Badenhorst C.P.S., Van Der Sluis R., Erasmus E., Van Dijk A.A. (2013). Glycine Conjugation: Importance in Metabolism, the Role of Glycine *N*-Acyltransferase, and Factors That Influence Interindividual Variation. Expert Opin. Drug Metab. Toxicol..

[61] Tian X., Wu L., Jiang M., Zhang Z., Wu R., Miao J., Liu C., Gao S. (2021). Downregulation of GLYAT Facilitates Tumor Growth and Metastasis and Poor Clinical Outcomes Through the PI3K/AKT/Snail Pathway in Human Breast Cancer. Front. Oncol..

[62] Xia Y., Huang W., Jin G.-Z. (2024). GLYAT Suppresses Liver Cancer and Clear Cell Renal Cell Carcinoma Progression by Downregulating ROCK1 Expression. Transl. Cancer Res..

[63] Cengiz B., Yumrutas O., Bozgeyik E., Borazan E., Igci Y.Z., Bozgeyik I., Oztuzcu S. (2015). Differential Expression of the UGT1A Family of Genes in Stomach Cancer Tissues. Tumor Biol..

[64] Maruo Y., Iwai M., Mori A., Sato H., Takeuchi Y. (2005). Polymorphism of UDP-Glucuronosyltransferase and Drug Metabolism. CDM.

[65] Bussey K.J., Davies P.C.W. (2021). Reverting to Single-Cell Biology: The Predictions of the Atavism Theory of Cancer. Prog. Biophys. Mol. Biol..

[66] Chen H., Lin F., Xing K., He X. (2015). The Reverse Evolution from Multicellularity to Unicellularity during Carcinogenesis. Nat. Commun..

[67] Thomas F., Ujvari B., Renaud F., Vincent M. (2017). Cancer Adaptations: Atavism, de Novo Selection, or Something in Between?. BioEssays.

[68] Vincent M. (2012). Cancer: A De-repression of a Default Survival Program Common to All Cells?: A Life-history Perspective on the Nature of Cancer. BioEssays.

[69] Lambert G., Estévez-Salmeron L., Oh S., Liao D., Emerson B.M., Tlsty T.D., Austin R.H. (2011). An Analogy between the Evolution of Drug Resistance in Bacterial Communities and Malignant Tissues. Nat. Rev. Cancer.

[70] Przybycinski J., Nalewajska M., Marchelek-Mysliwiec M., Dziedziejko V., Pawlik A. (2019). Poly-ADP-Ribose Polymerases (PARPs) as a Therapeutic Target in the Treatment of Selected Cancers. Expert Opin. Ther. Targets.

[71] Zhao X., Zhu Y., Hu J., Jiang L., Li L., Jia S., Zen K. (2018). Shikonin Inhibits Tumor Growth in Mice by Suppressing Pyruvate Kinase M2-Mediated Aerobic Glycolysis. Sci. Rep..

[72] Le D.T., Durham J.N., Smith K.N., Wang H., Bartlett B.R., Aulakh L.K., Lu S., Kemberling H., Wilt C., Luber B.S. (2017). Mismatch Repair Deficiency Predicts Response of Solid Tumors to PD-1 Blockade. Science.

[73] Garcia D., Shaw R.J. (2017). AMPK: Mechanisms of Cellular Energy Sensing and Restoration of Metabolic Balance. Mol. Cell.

[74] Wrenn E., Huang Y., Cheung K. (2021). Collective Metastasis: Coordinating the Multicellular Voyage. Clin. Exp. Metastasis.

[75] Asano K., Nelson C.M., Nandadasa S., Aramaki-Hattori N., Lindner D.J., Alban T., Inagaki J., Ohtsuki T., Oohashi T., Apte S.S. (2017). Stromal Versican Regulates Tumor Growth by Promoting Angiogenesis. Sci. Rep..

[76] Chang A.C.-M., Doherty J., Huschtscha L.I., Redvers R., Restall C., Reddel R.R., Anderson R.L. (2015). STC1 Expression Is Associated with Tumor Growth and Metastasis in Breast Cancer. Clin. Exp. Metastasis.

[77] Cheng Y., Sun H., Wu L., Wu F., Tang W., Wang X., Lv C. (2020). VUp-Regulation of VCAN Promotes the Proliferation, Invasion and Migration and Serves as a Biomarker in Gastric Cancer. OTT.

[78] Ghosh S., Albitar L., LeBaron R., Welch W.R., Samimi G., Birrer M.J., Berkowitz R.S., Mok S.C. (2010). Up-Regulation of Stromal Versican Expression in Advanced Stage Serous Ovarian Cancer. Gynecol. Oncol..

[79] He L., Wang T., Gao Q., Zhao G., Huang Y., Yu L., Hou Y. (2011). Stanniocalcin-1 Promotes Tumor Angiogenesis through up-Regulation of VEGF in Gastric Cancer Cells. J. Biomed. Sci..

[80] Law A.Y.S., Wong C.K.C. (2013). Stanniocalcin-1 and -2 Promote Angiogenic Sprouting in HUVECs via VEGF/VEGFR2 and Angiopoietin Signaling Pathways. Mol. Cell. Endocrinol..

[81] Xu C., Sun L., Jiang C., Zhou H., Gu L., Liu Y., Xu Q. (2017). SPP1, Analyzed by Bioinformatics Methods, Promotes the Metastasis in Colorectal Cancer by Activating EMT Pathway. Biomed. Pharmacother..

[82] Zeng B., Zhou M., Wu H., Xiong Z. (2018). SPP1 Promotes Ovarian Cancer Progression via Integrin β1/FAK/Akt Signaling Pathway. OncoTargets Ther..

[83] Jain S., Raza K., Agrawal A.K., Vaidya A. (2021). Therapy Targeting Angiogenic Potential of Tumor. Nanotechnology Applications for Cancer Chemotherapy.

[84] Lin X., Kang K., Chen P., Zeng Z., Li G., Xiong W., Yi M., Xiang B. (2024). Regulatory Mechanisms of PD-1/PD-L1 in Cancers. Mol. Cancer.

[85] Amin M.B., Greene F.L., Edge S.B., Compton C.C., Gershenwald J.E., Brookland R.K., Meyer L., Gress D.M., Byrd D.R., Winchester D.P. (2017). The Eighth Edition AJCC Cancer Staging Manual: Continuing to Build a Bridge from a Population-based to a More “Personalized” Approach to Cancer Staging. CA A Cancer J. Clin..

[86] Aran D., Camarda R., Odegaard J., Paik H., Oskotsky B., Krings G., Goga A., Sirota M., Butte A.J. (2017). Comprehensive Analysis of Normal Adjacent to Tumor Transcriptomes. Nat. Commun..

[87] Chandran U.R., Dhir R., Ma C., Michalopoulos G., Becich M., Gilbertson J. (2005). Differences in Gene Expression in Prostate Cancer, Normal Appearing Prostate Tissue Adjacent to Cancer and Prostate Tissue from Cancer Free Organ Donors. BMC Cancer.

[88] Sanz-Pamplona R., Berenguer A., Cordero D., Molleví D.G., Crous-Bou M., Sole X., Paré-Brunet L., Guino E., Salazar R., Santos C. (2014). Aberrant Gene Expression in Mucosa Adjacent to Tumor Reveals a Molecular Crosstalk in Colon Cancer. Mol. Cancer.

[89] Huang X., Stern D.F., Zhao H. (2016). Transcriptional Profiles from Paired Normal Samples Offer Complementary Information on Cancer Patient Survival—Evidence from TCGA Pan-Cancer Data. Sci. Rep..

[90] Ritchie M.E., Phipson B., Wu D., Hu Y., Law C.W., Shi W., Smyth G.K. (2015). Limma Powers Differential Expression Analyses for RNA-Sequencing and Microarray Studies. Nucleic Acids Res..

[91] Mustafin Z.S., Lashin S.A., Matushkin Y.G. (2021). Phylostratigraphic Analysis of Gene Networks of Human Diseases. Vavilov J. Genet. Breed..

[92] Ivanov R.A., Mukhin A.M., Kazantsev F.V., Mustafin Z.S., Afonnikov D.A., Matushkin Y.G., Lashin S.A. (2025). Orthoweb: A Software Package for Evolutionary Analysis of Gene Networks. Vavilov J. Genet. Breed..

[93] Kanehisa M., Sato Y., Kawashima M., Furumichi M., Tanabe M. (2016). KEGG as a Reference Resource for Gene and Protein Annotation. Nucleic Acids Res..

[94] Waskom M.L. (2021). seaborn: Statistical data visualization. J. Open Source Softw..

