# Supplementary material for: Evolutionary Transcriptomics of Cancer Development
Source: Int J Mol Sci. 2025 May 23;26(11):5041. doi: 10.3390/ijms26115041 (PMC12155295; doi:10.3390/ijms26115041)
Supplement: Supplementary file 1 [file ijms-26-05041-s001.zip › ijms-3620054-supplementary.pdf]

Supplementary Table S1 Transcriptome Age Index values across clinical stages of cancers and statistical significance of the “reductive hourglass” and “reverse hourglass” tests

| TCGA_project | NAT | Stage_I | Stage_II | Stage_III | Stage_IV | p_value |
|---|---|---|---|---|---|---|
| Significant "hourglass" pattern in TAI distribution by clinical stages | | | | | | p_rht |
| LIHC | 5.99863 | 5.403328 | 5.32169 | 5.192634 | 5.459084 | 0.0144 |
| BLCA | 4.581481 | 4.542902 | 4.492966 | 4.505613 | 4.534112 | 0.0153 |
| BRCA | 4.785891 | 4.668032 | 4.637815 | 4.665382 | 4.677226 | 0.0115 |
| Significant "reverse hourglass" pattern in TAI distribution by clinical stages | | | | | | p_reverse_hourglass |
| COAD | 4.850936 | 4.439789 | 4.45038 | 4.481497 | 4.456311 | 0.0374 |
| KIRC | 4.798397 | 4.853467 | 4.862405 | 4.863008 | 4.825348 | 0.00684 |
| No significant patterns in TAI distribution | | | | | | |
| LUAD | 5.310213 | 4.784176 | 4.733831 | 4.706017 | 4.703828 | 0.899 |
| PRAD | 4.742203 | 4.712207 | 4.675865 | 4.675532 | 4.662134 | 0.41 |
| THCA | 4.972783 | 4.843374 | 4.828472 | 4.828682 | 4.831671 | 0.291 |
| UCEC | 4.607078 | 4.463704 | 4.467779 | 4.445552 | 4.455624 | 0.308 |

Supplementary Table S2 Transcriptome Divergence Index values across clinical stages of cancers and statistical significance of the “reductive hourglass” and tests

| TCGA_project | NAT | Stage_I | Stage_II | Stage_III | Stage_IV | p_value |
|---|---|---|---|---|---|---|
| Significant "hourglass" pattern in TDI distribution by clinical stages | | | | | | p_rht |
| LUAD | 4.734863 | 4.254778 | 4.200492 | 4.170093 | 4.194738 | 0.0423 |
| PRAD | 4.499775 | 4.274551 | 4.243194 | 4.250399 | 4.343177 | 0.017 |
| No significant patterns in TDI distribution | | | | | | |
| UCEC | 3.967398 | 3.986911 | 3.998328 | 3.971333 | 4.001767 | 0.188 |
| BLCA | 3.984667 | 4.257497 | 4.048051 | 4.004898 | 4.011184 | 0.464 |
| BRCA | 4.190287 | 4.078578 | 4.069145 | 4.073497 | 4.095158 | 0.0862 |
| COAD | 4.495807 | 4.066575 | 4.071877 | 4.074385 | 4.065866 | 0.0898 |
| KIRC | 4.275209 | 4.247558 | 4.242292 | 4.243964 | 4.223329 | 0.503 |
| THCA | 4.520441 | 4.244759 | 4.301416 | 4.204916 | 4.178473 | 0.88 |
| LIHC | 5.571663 | 5.097211 | 5.24053 | 4.864015 | 5.187719 | 0.203 |

Supplementary Table S3. List of investigated Gene Ontology (GO) terms associated with processes occurring in malignant tumors.

| GO ID | GO term |
|---|---|
| GO:0051726 | regulation of cell cycle |
| GO:0000082 | G1/S transition of mitotic cell cycle |
| GO:0000086 | G2/M transition of mitotic cell cycle |
| GO:0007088 | regulation of mitotic nuclear division |
| GO:0042127 | regulation of cell population proliferation |
| GO:0071774 | response to fibroblast growth factor |
| GO:0031577 | spindle checkpoint signaling |
| GO:0006260 | DNA replication |
| GO:0051276 | chromosome organization |
| GO:0032200 | telomere organization |

| | |
|---|---|
| GO:0000723 | telomere maintenance |
| GO:0030154 | cell differentiation |
| GO:0006399 | tRNA metabolic process |
| GO:0006397 | mRNA processing |
| GO:0006259 | DNA metabolic process |
| GO:0006412 | translation |
| GO:0006915 | apoptotic process |
| GO:0012501 | programmed cell death |
| GO:0006914 | autophagy |
| GO:0043066 | negative regulation of apoptotic process |
| GO:0043065 | positive regulation of apoptotic process |
| GO:0006351 | DNA-templated transcription |
| GO:0016055 | Wnt signaling pathway |
| GO:0000165 | MAPK cascade |
| GO:0014065 | phosphatidylinositol 3-kinase signaling |
| GO:0007179 | transforming growth factor beta receptor signaling pathway |
| GO:0010629 | negative regulation of gene expression |
| GO:0045893 | positive regulation of DNA-templated transcription |
| GO:0040029 | epigenetic regulation of gene expression |
| GO:0006281 | DNA repair |
| GO:0006302 | double-strand break repair |
| GO:0006303 | double-strand break repair via nonhomologous end joining |
| GO:0006974 | DNA damage response |
| GO:0006096 | glycolytic process |
| GO:0006099 | tricarboxylic acid cycle |
| GO:0006635 | fatty acid beta-oxidation |
| GO:0009058 | biosynthetic process |
| GO:0008203 | cholesterol metabolic process |
| GO:0016477 | cell migration |
| GO:0060326 | cell chemotaxis |
| GO:2000145 | regulation of cell motility |
| GO:0001525 | angiogenesis |
| GO:0001568 | blood vessel development |
| GO:0001935 | endothelial cell proliferation |
| GO:0048870 | cell motility |
| GO:0007155 | cell adhesion |
| GO:0007160 | cell-matrix adhesion |
| GO:0006954 | inflammatory response |
| GO:0007267 | cell-cell signaling |
| GO:0019882 | antigen processing and presentation |
| GO:0002250 | adaptive immune response |
| GO:0006955 | immune response |
| GO:0002418 | immune response to tumor cell |

Violin plots of Log2FC distributions by PAI for differentially expressed genes in tumor tissues across pathological stages

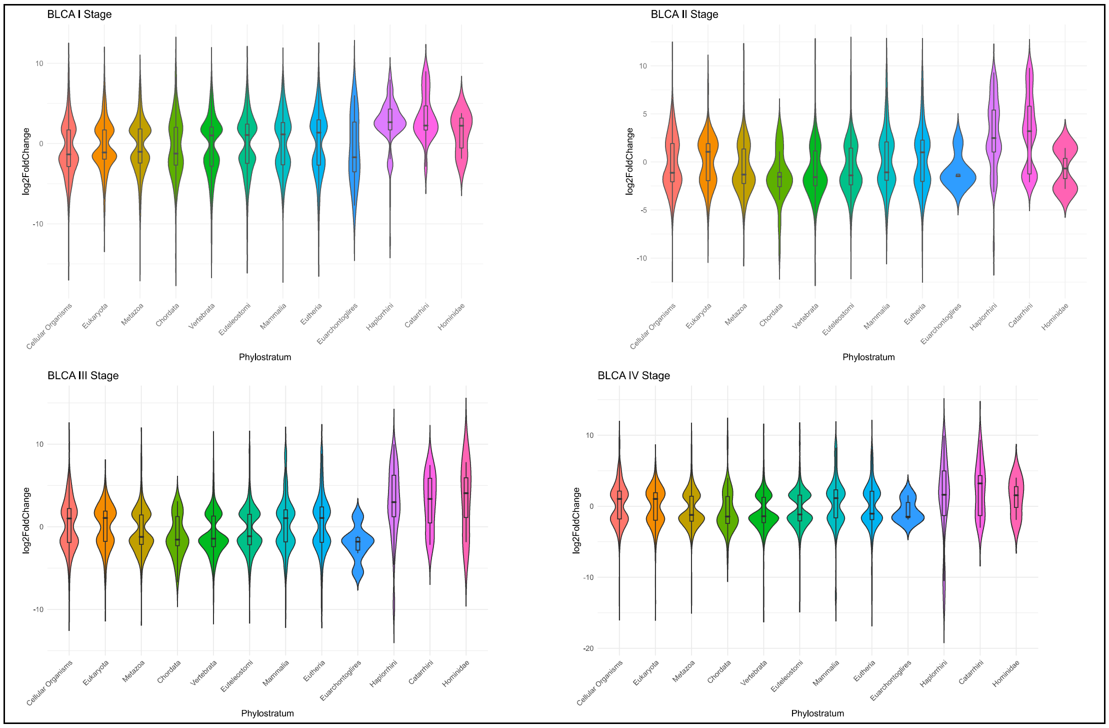


Figure S1. Violin plots of log2FC distributions for differentially expressed genes (DEGs) by phylostratigraphic indices in bladder carcinoma (BLCA). A shift from negative median values in PAI=1–2 at stage I to positive values at stage IV is observed.

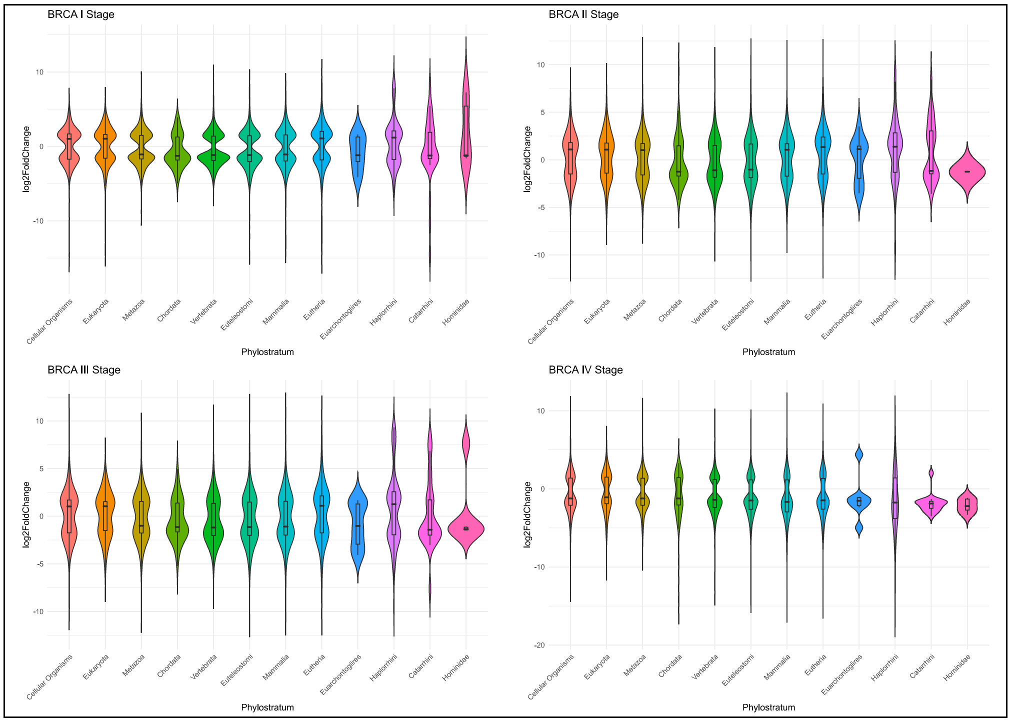


Figure S2. Violin plots of log2FC distributions for DEGs by phylostratigraphic indices in breast ductal carcinoma (BRCA). A sharp decline in log2FC values for ranks 1–2 and 10–14 is noted at stage IV.

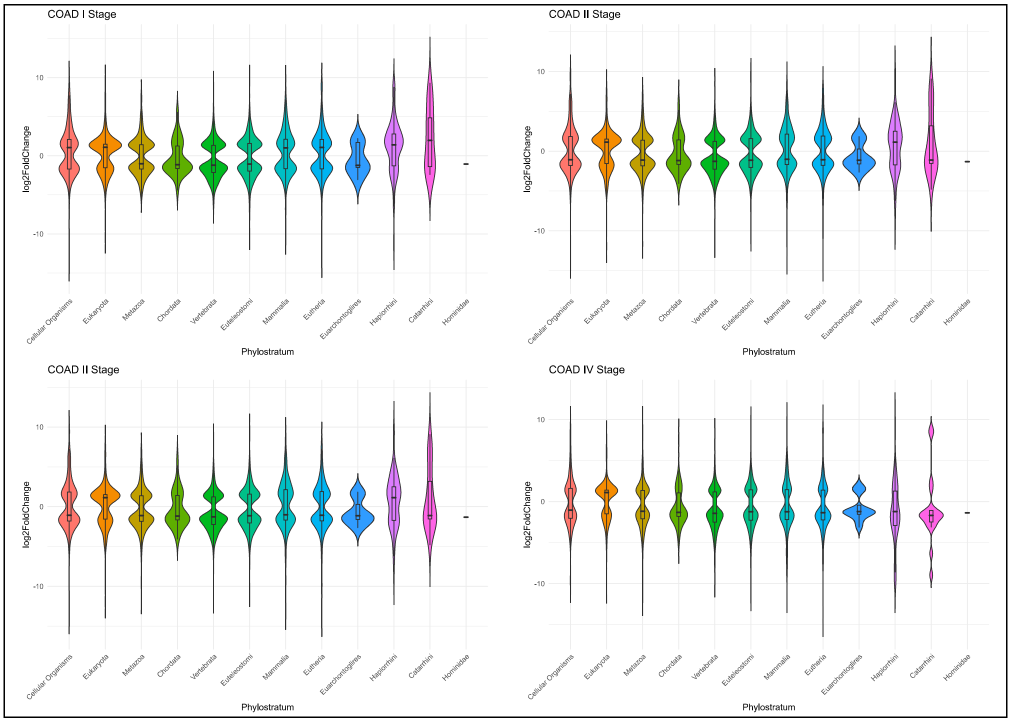

Figure S3. Violin plots of log2FC distributions for DEGs by phylostratigraphic indices in colorectal adenocarcinoma (COAD).

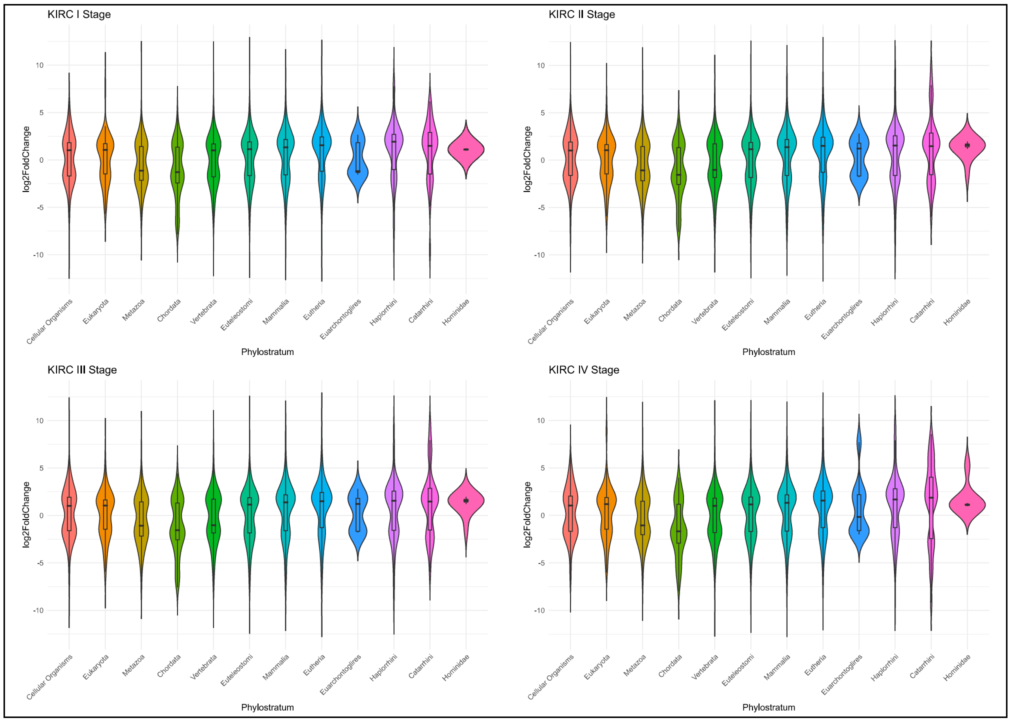

Figure S4. Violin plots of log2FC distributions for DEGs by phylostratigraphic indices in clear cell renal carcinoma (KIRC). Consistently negative median log2FC values for genes with PAI=3, 4 across all stages.

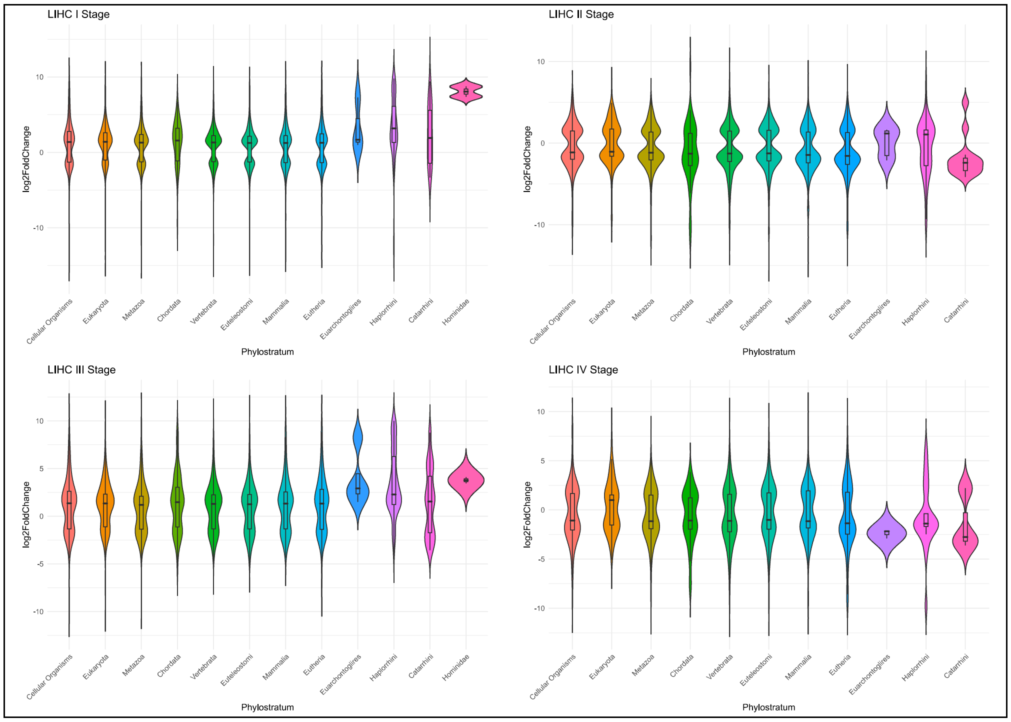


Figure S5. Violin plots of log2FC distributions for DEGs by phylostratigraphic indices in hepatocellular carcinoma (LIHC). High positive median log2FC values at stage I transition to extremely negative values at stage IV.

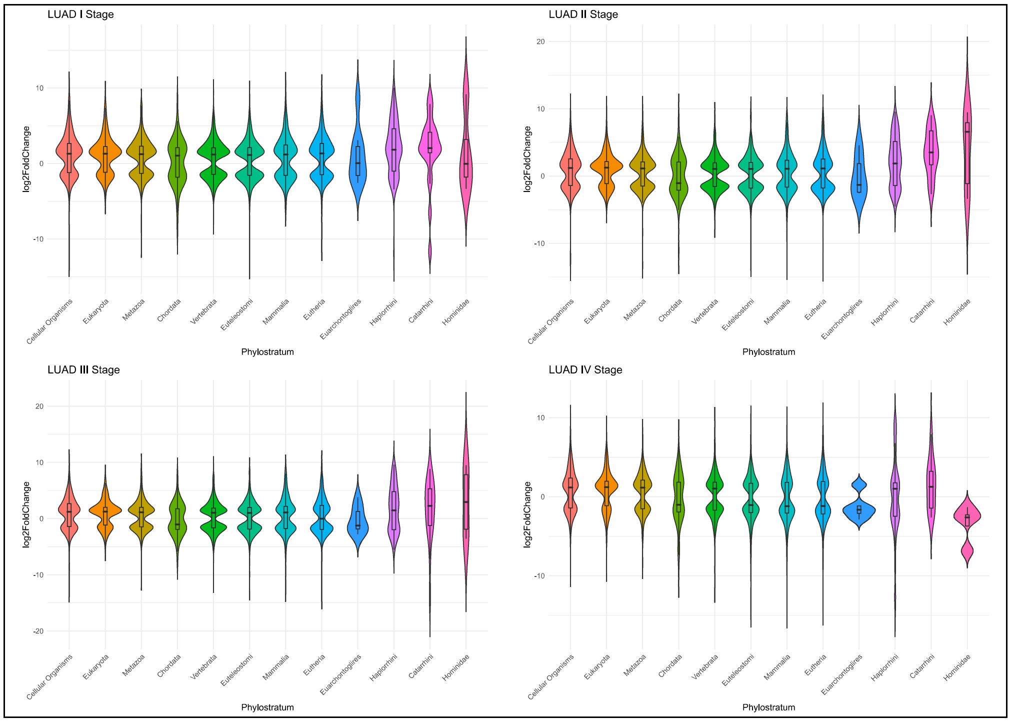


Figure S6. Violin plots of log2FC distributions for DEGs by phylostratigraphic indices in lung adenocarcinoma (LUAD). Positive peaks in PAI=7–10 at stages II–III sharply decline at stage IV.

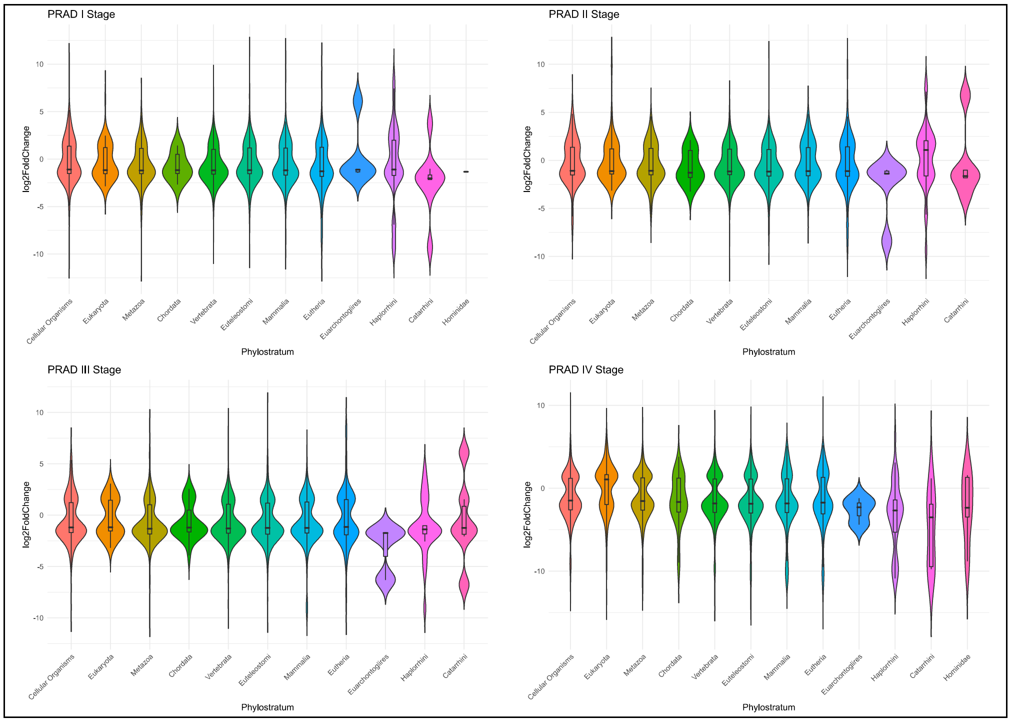


Figure S7. Violin plots of log2FC distributions for DEGs by phylostratigraphic indices in prostate adenocarcinoma (PRAD). Consistently negative median log2FC values across all stages.

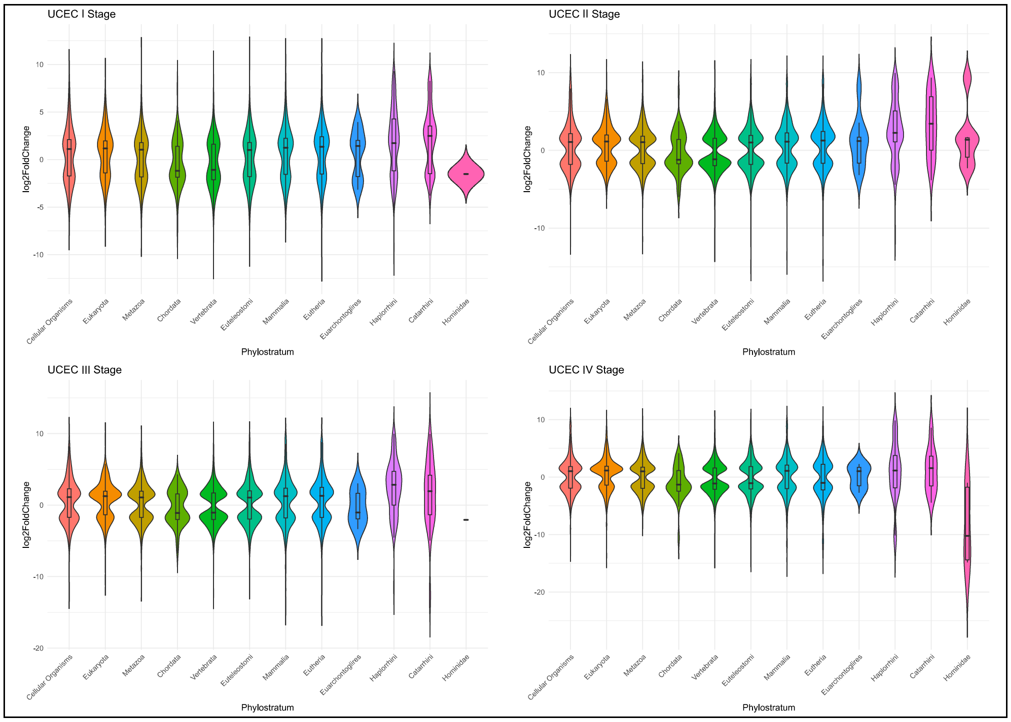


Figure S8. Violin plots of log2FC distributions for DEGs by phylostratigraphic indices in uterine corpus carcinoma (UCEC). Persistent negative median log2FC values in phylostrata 4 and 6 (Chordata and Vertebrata ) across all stages.

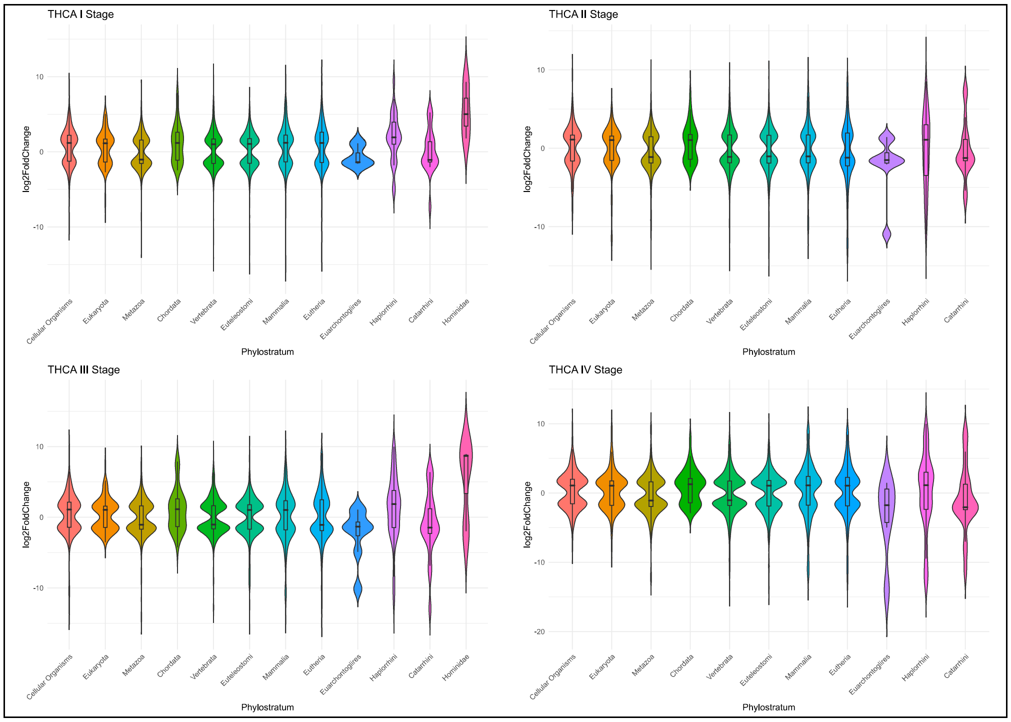


Figure S9. Violin plots of log2FC distributions for DEGs by phylostratigraphic indices in thyroid carcinoma (THCA). Consistently positive median log2FC values in phylostrata 1, 2, 4 (Cellular Organism , Eukaryote , Chordata ) and negative median log2FC in phylostratum 3 (Metazoa ) across all stages.

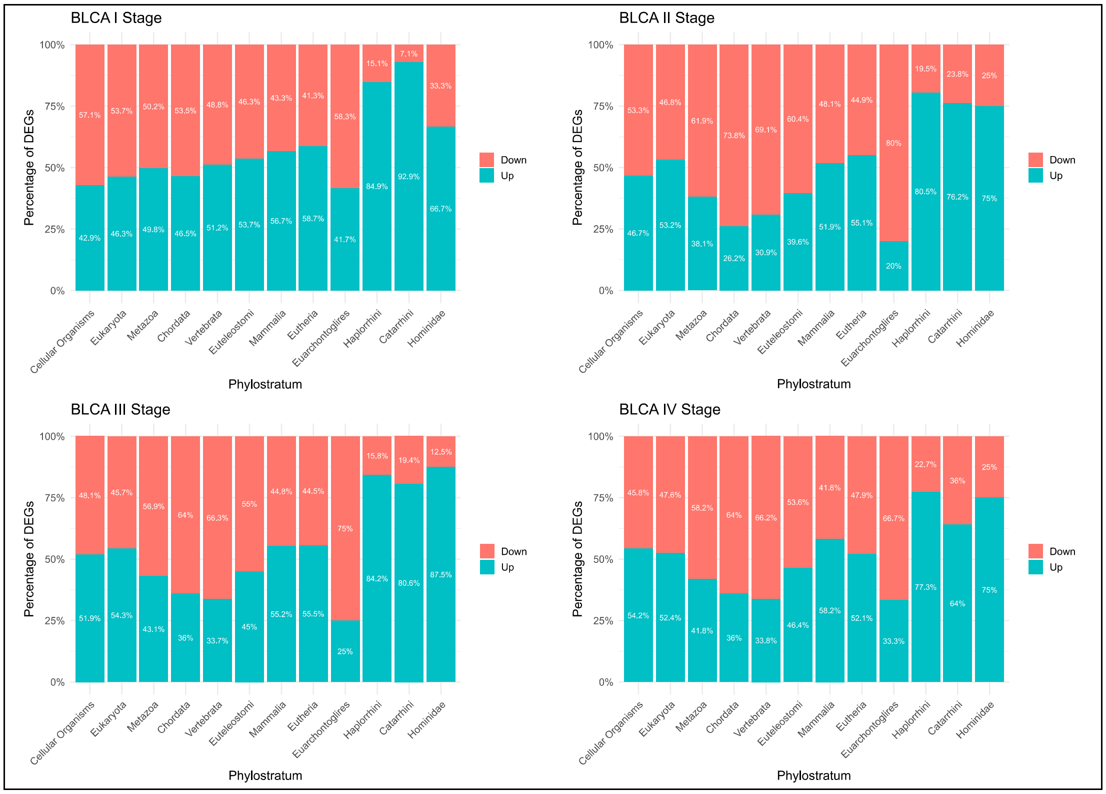


Figure S10. Bar charts of percentage ratios of up- and down-regulated genes for different PAI values in bladder carcinoma (BLCA) across stages.

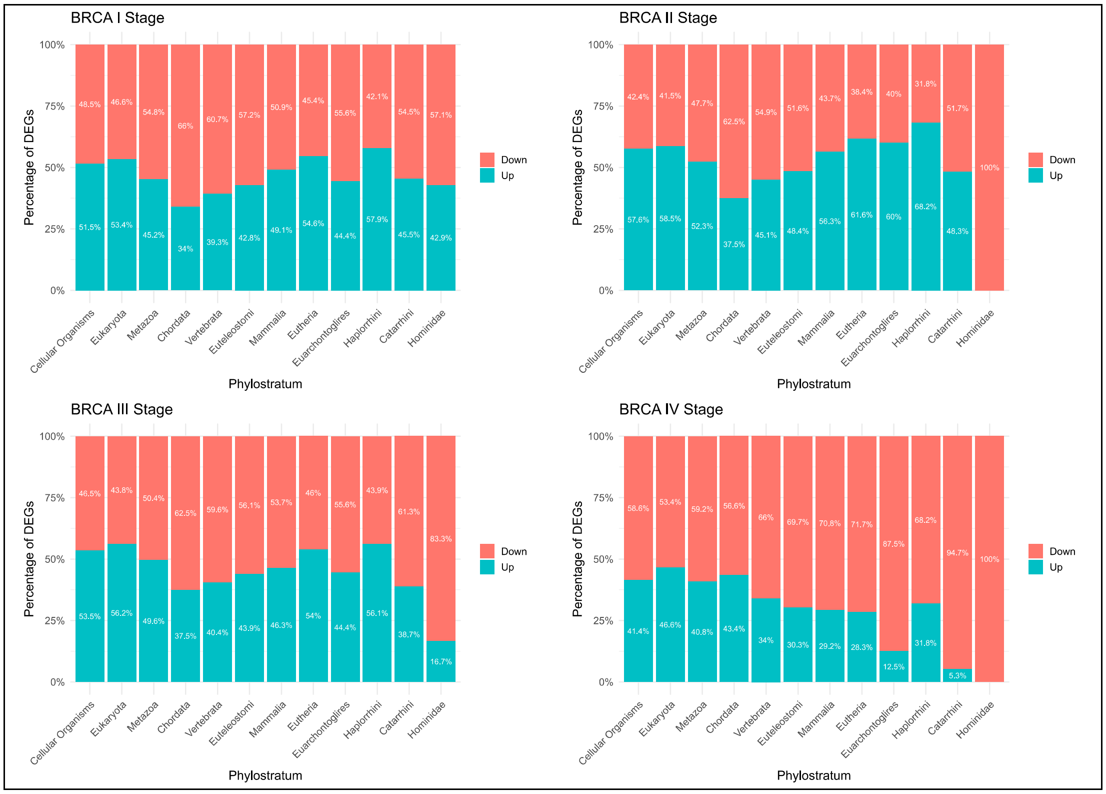


Figure S11. Bar charts of percentage ratios of up- and down-regulated genes for different PAI values in breast ductal carcinoma (BRCA) across stages.

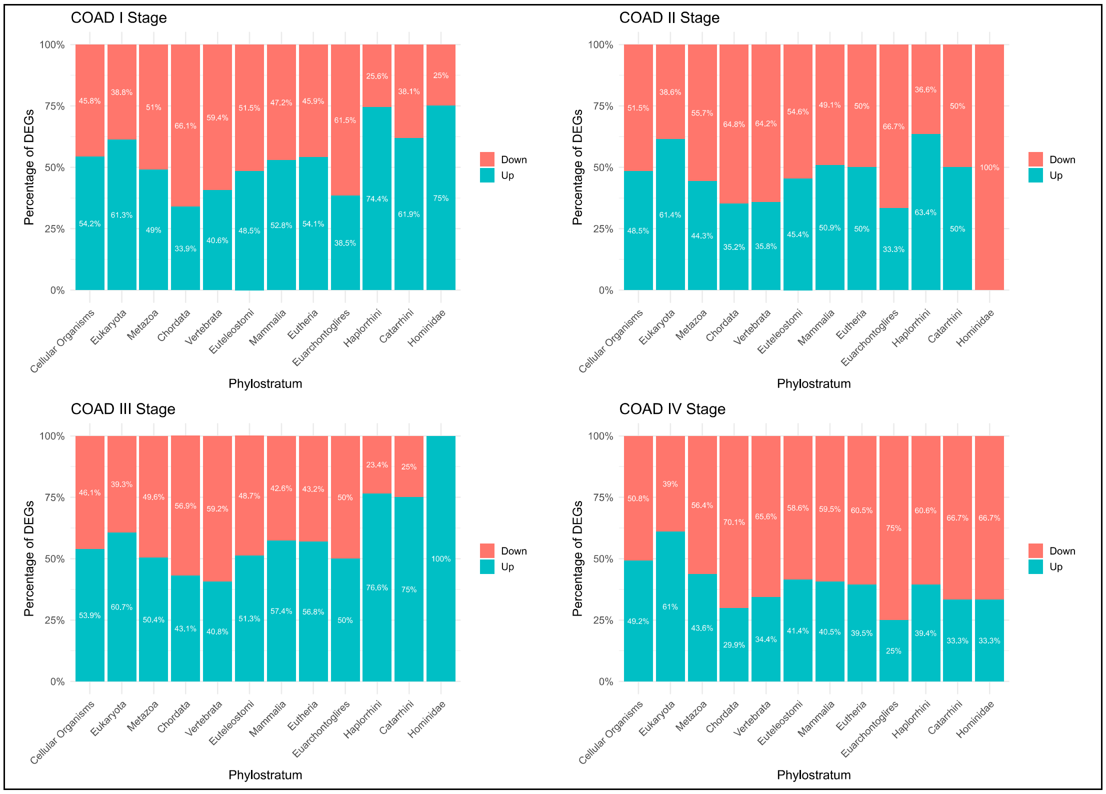


Figure S12. Bar charts of percentage ratios of up- and down-regulated genes for different PAI values in colorectal adenocarcinoma (COAD) across stages.

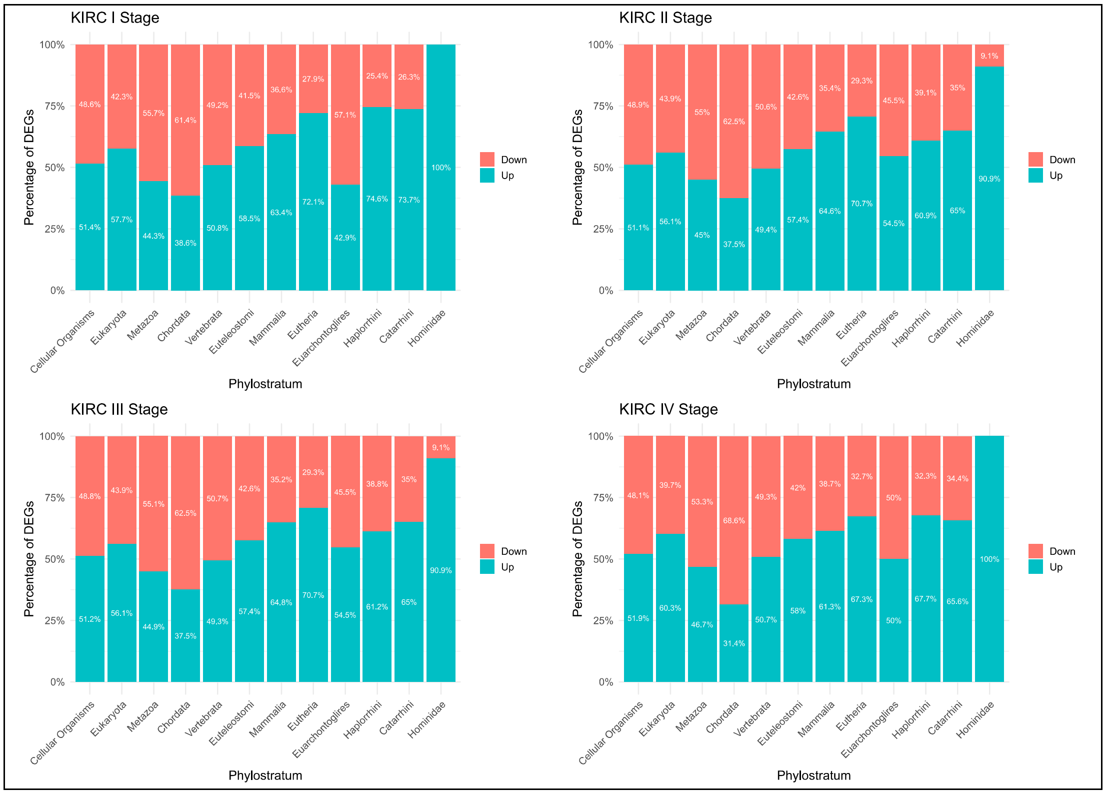


Figure S13. Bar charts of percentage ratios of up- and down-regulated genes for different PAI values in clear cell renal carcinoma (KIRC) across stages

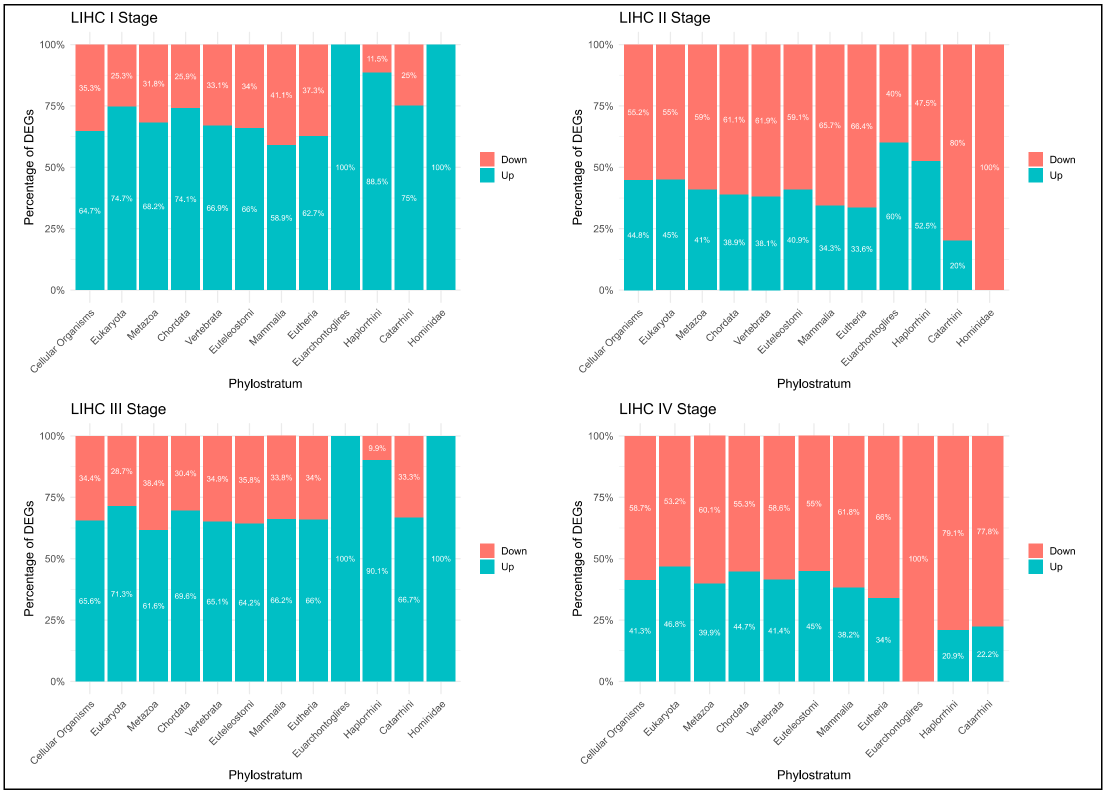


Figure S14. Bar charts of percentage ratios of up- and down-regulated genes for different PAI values in hepatocellular carcinoma (LIHC) across stages.

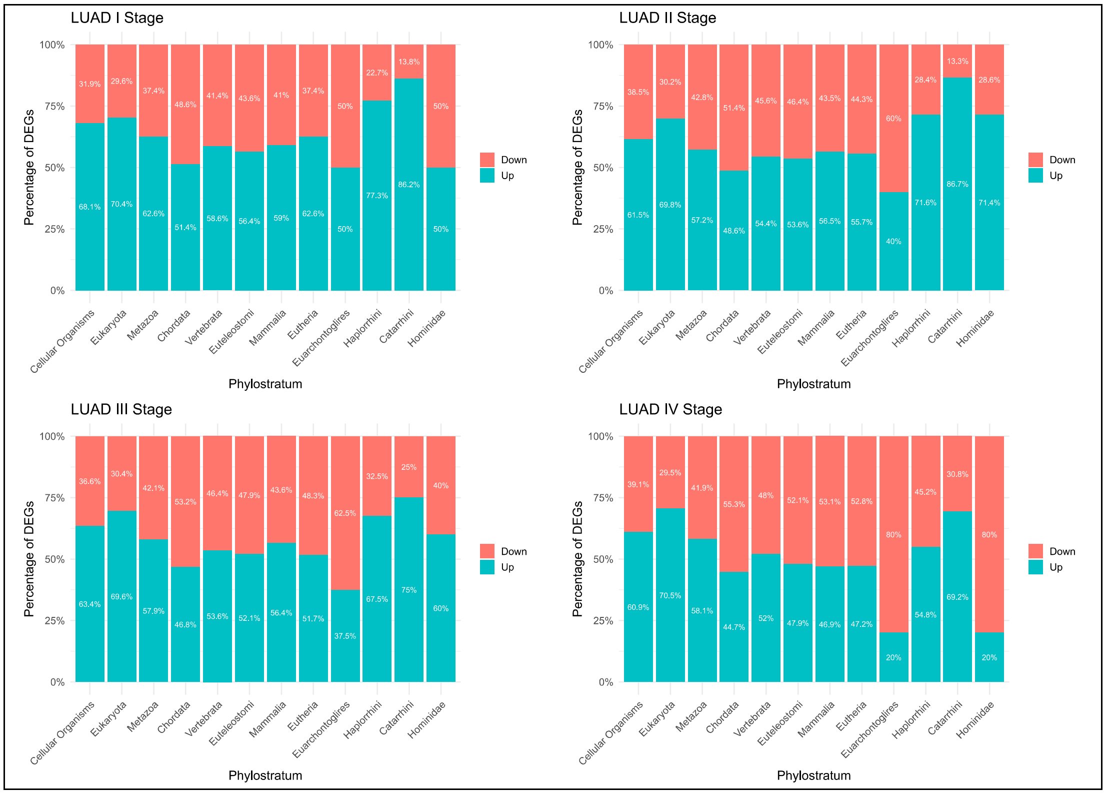


Figure S15. Bar charts of percentage ratios of up- and down-regulated genes for different PAI values in lung adenocarcinoma (LUAD) across stages.

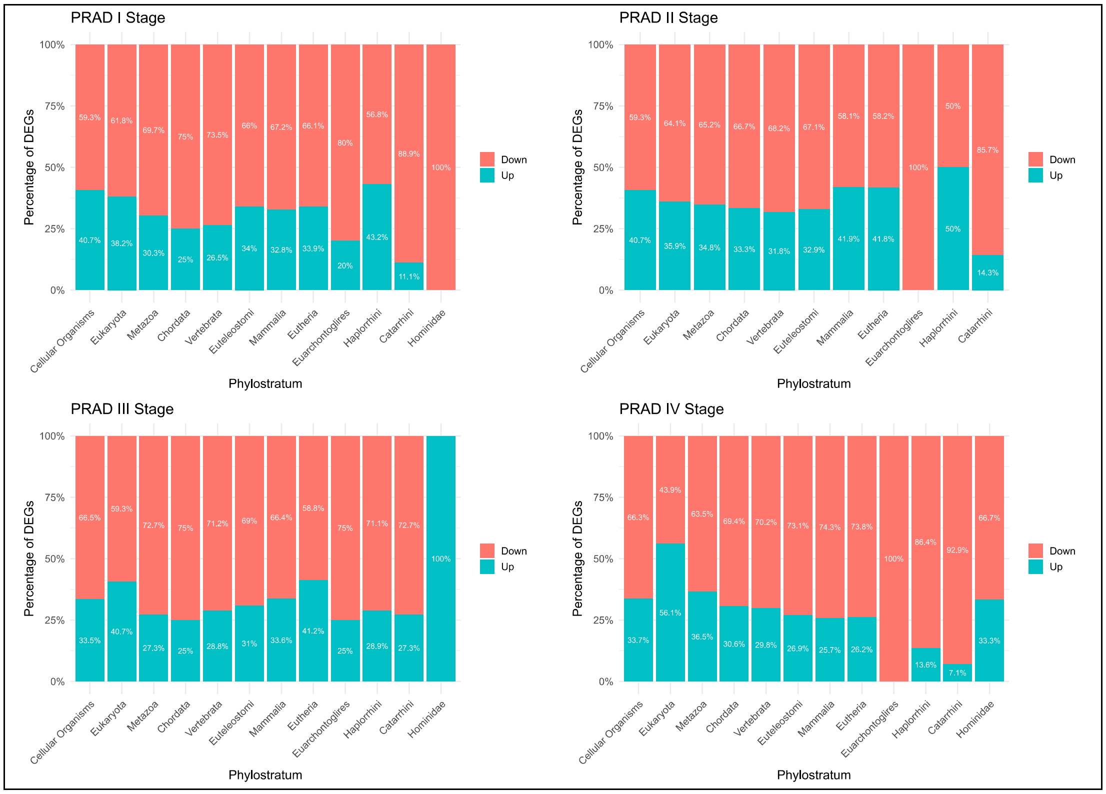


Figure S16. Bar charts of percentage ratios of up- and down-regulated genes for different PAI values in prostate adenocarcinoma (PRAD) across stages.

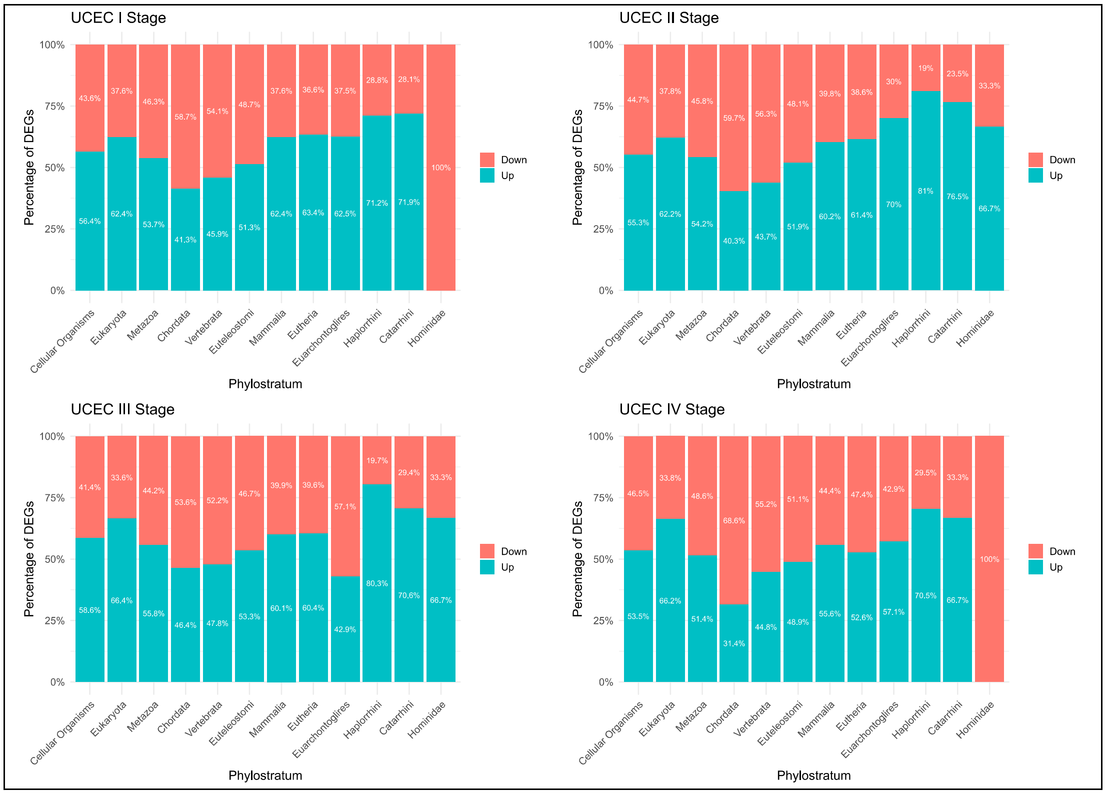


Figure S17. Bar charts of percentage ratios of up- and down-regulated genes for different PAI values in uterine corpus carcinoma (UCEC) across stages.

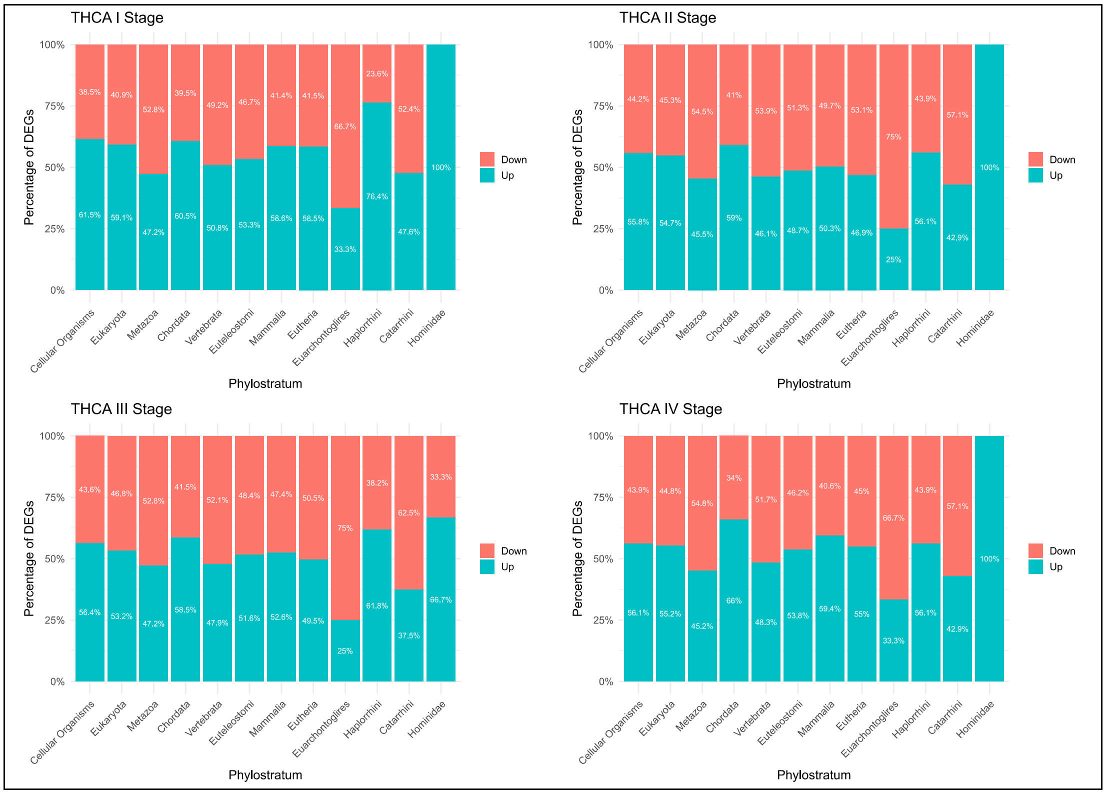


Figure S18. Bar charts of percentage ratios of up- and down-regulated genes for different PAI values in thyroid carcinoma (THCA) across stages.
